# Identification of distinct miRNA target regulation between breast cancer molecular subtypes using AGO2-PAR-CLIP and patient datasets

**DOI:** 10.1186/gb-2014-15-1-r9

**Published:** 2014-01-07

**Authors:** Thalia A Farazi, Jelle J ten Hoeve, Miguel Brown, Aleksandra Mihailovic, Hugo M Horlings, Marc J van de Vijver, Thomas Tuschl, Lodewyk FA Wessels

**Affiliations:** 1 Howard Hughes Medical Institute, Laboratory of RNA Molecular Biology, The Rockefeller University, 1230 York Avenue, Box 186, New York, NY 10065 USA; 2 Division of Molecular Carcinogenesis, The Netherlands Cancer Institute, Plesmanlaan 121, 1066 CX Amsterdam, The Netherlands; 3 Department of Pathology, Academic Medical Center, Meibergdreef 9, 1105 AZ Amsterdam, The Netherlands; 4 CancerGenomics.nl

## Abstract

**Background:**

Various microRNAs (miRNAs) are up- or downregulated in tumors. However, the repression of cognate miRNA targets responsible for the phenotypic effects of this dysregulation in patients remains largely unexplored. To define miRNA targets and associated pathways, together with their relationship to outcome in breast cancer, we integrated patient-paired miRNA-mRNA expression data with a set of validated miRNA targets and pathway inference.

**Results:**

To generate a biochemically-validated set of miRNA-binding sites, we performed argonaute-2 photoactivatable-ribonucleoside-enhanced crosslinking and immunoprecipitation (AGO2-PAR-CLIP) in MCF7 cells. We then defined putative miRNA-target interactions using a computational model, which ranked and selected additional TargetScan-predicted interactions based on features of our AGO2-PAR-CLIP binding-site data. We subselected modeled interactions according to the abundance of their constituent miRNA and mRNA transcripts in tumors, and we took advantage of the variability of miRNA expression within molecular subtypes to detect miRNA repression. Interestingly, our data suggest that miRNA families control subtype-specific pathways; for example, miR-17, miR-19a, miR-25, and miR-200b show high miRNA regulatory activity in the triple-negative, basal-like subtype, whereas miR-22 and miR-24 do so in the HER2 subtype. An independent dataset validated our findings for miR-17 and miR-25, and showed a correlation between the expression levels of miR-182 targets and overall patient survival. Pathway analysis associated miR-17, miR-19a, and miR-200b with leukocyte transendothelial migration.

**Conclusions:**

We combined PAR-CLIP data with patient expression data to predict regulatory miRNAs, revealing potential therapeutic targets and prognostic markers in breast cancer.

## Background

Breast cancer is a heterogeneous disease involving various tumorigenesis mechanisms manifesting at the DNA, RNA, and protein level. Patients are classified by estrogen receptor (ESR/ER), progesterone receptor (PGR/PR), and ERBB2*/*HER2 amplified oncogene expression based on immunohistochemistry, molecular subtypes based on mRNA expression signatures (luminal, basal-like, HER2, normal-like), or integrated clusters based on combination of mRNA expression and DNA copy number alteration [1]. Prognostic mRNA expression signatures have been defined for specific sets of breast tumors [2,3], but given the heterogeneity of patient outcomes within the same subtype, it is clear that pathways regulating tumor aggressiveness remain to be further elucidated. miRNAs have shown promise as therapeutic targets in cancer, suggested by the recent introduction of the first miRNA mimic in Phase I cancer clinical trials, and as diagnostic/prognostic markers, suggested by their cell-type specificity. Oncogenic and tumor suppressive miRNAs have been implicated in the regulation of critical cellular pathways, such as differentiation and apoptosis, across several tumor types [4-6], but identifying miRNA target regulation/repression in tumor samples remains challenging.

Multiple studies have examined the correlation between miRNA and mRNA expression in breast tumors as well as the role of miRNA expression in prognosis, using samples from variable molecular subtypes, but a clear conclusion has yet to be reached (Additional file 1: Table S1) [7-12]. The Cancer Genome Atlas (TCGA) published same-sample miRNA and mRNA expression profiles for a large patient collection (*n* = 797) determined by sequencing but has not commented on miRNA targeting activity and prognosis [13]. Finally, a recent study including 1,302 breast tumors, utilizing miRNA and mRNA expression by microarrays, did not determine direct miRNA target repression [14]. The variability of findings, some of which is due to technical limitations of quantification methods, highlights the need for further studies and detailed examination of approaches used for correlation analysis aimed at establishing regulatory relationships between miRNAs and their targets in patient samples.

**Figure 1:**
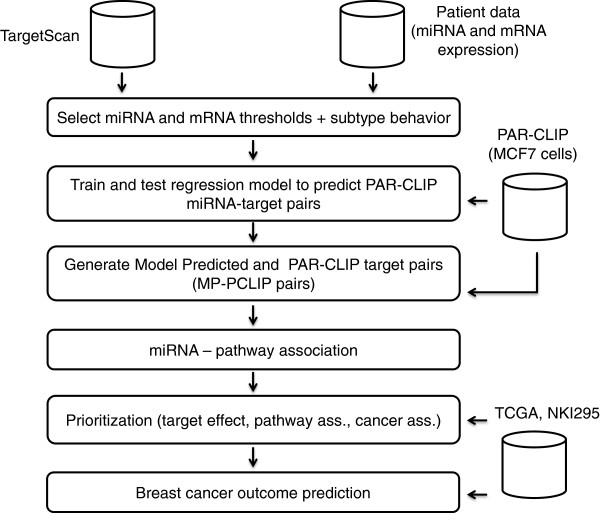
Overview of analysis.

We recently reported miRNA profiles of a well-characterized breast cancer collection (*n* = 179) using small RNA cDNA library preparation and deep sequencing, with 161 of these also studied using mRNA microarrays [15]. Here, we used the patient miRNA and mRNA expression profiles, TargetScan predictions [16] and AGO2-PAR-CLIP [17] to identify miRNA targets (Figure 1). First, we selected miRNAs and mRNAs from the patient data based on their expression levels and conducted the analysis within molecular subtypes. Our study differs from earlier studies in that it includes miRNA binding sites determined experimentally by AGO2-PAR-CLIP in ductal MCF7 cells. We defined a list of validated miRNA-target interactions by using the experimentally supported AGO2-PAR-CLIP interactions and training a regression model to rank and select miRNA target interactions from TargetScan predictions that display similar characteristics to AGO2-PAR-CLIP targets. We then prioritized miRNA regulatory activity based on association with expression of respective validated targets, as well as association with KEGG pathways and known cancer genes. Finally, we predicted outcome among molecular subtypes based on miRNA and respective target expression. We validated and compared our results in two independent datasets: TCGA [13] and NKI295 [3]. We provide the prioritization of miRNA targets, miRNA pathway association, and miRNA activity in a web-based format that can be easily sorted for molecular subtype and dataset, and searched for a particular miRNA, mRNA target, and pathway [18].

## Results

### Correlations between miRNA families and their targets depend on mRNA and miRNA abundance

We conducted correlation analysis of the same-sample miRNA-mRNA expression from 161 patient samples from our earlier study [15], and a selection of 444 samples from the TCGA study [13]. Our samples included normal breast, ductal carcinoma in situ (DCIS), and invasive ductal carcinoma (IDC), comprising a variety of molecular subtypes. TCGA samples included invasive breast carcinomas also comprising a variety of molecular subtypes. In our dataset miRNA abundance was measured as relative read frequency (RRF) and mRNA abundance as the average fluorescence intensity from both channels of Operon arrays (A-value, see Materials and methods). In the TCGA dataset miRNA and mRNA expression levels were determined by sequencing; the miRNA abundance reported as RRF and mRNA abundance as reads per kilobase per million (RPKM). We confirmed that intronic miRNAs and their host protein-coding genes were positively correlated and established thresholds for miRNA abundance, selecting a threshold of 1e^-4^ RRF (see Materials and methods; Additional file 2: Figure S1 and S2).

To assess direct miRNA-target repression, we investigated whether correlations between expression of miRNAs with their computationally predicted-targets were more negative compared to all remaining miRNA-mRNA correlations, and explored whether mRNA abundance thresholds influenced the strength of the correlations. There are many miRNA target prediction algorithms, previously reviewed in depth [19-21]. TargetScan [16] and miRanda [22] demonstrated similar performance when evaluating the significance of enrichment of negative correlations between miRNAs and their targets in datasets from TCGA [23]. In addition to canonical miRNA targets defined by both algorithms, miRanda also determines non-canonical miRNA targets, computing a miRSVR score as the weighted sum of a number of sequence and context features of the predicted miRNA-mRNA duplex [22]. Our analysis showed that a larger set of conserved TargetScan-predicted targets performed similarly to a smaller set of stringent miRSVR scoring miRanda-predicted targets (Additional file 2: Figure S3) [22]. Thus, we chose to conduct our analysis using conserved TargetScan-predicted targets focusing on miRNA seed families to group miRNAs with similar regulatory potential. When we refer to miRNA correlations with their respective targets we refer to miRNA seed families as defined by TargetScan (referenced by the miRNA member of the lowest number).

**Figure 2:**
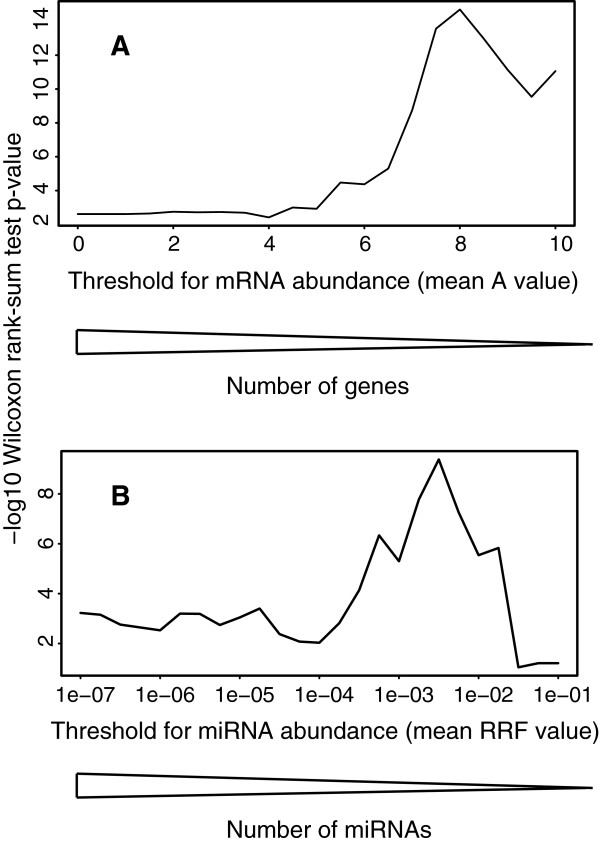
**MiRNA and mRNA abundance thresholds in patient datasets.** Dependence of Wilcoxon-rank-sum test *P* value of the difference of the medians of the distribution of miRNA-TargetScan-target correlations compared to the distribution of the remainder miRNA-mRNA correlations on selected threshold for mRNA **(A)** or miRNA abundance **(B)**. Results shown for all samples in [15].

Similarly to Dvinge et al., we did not observe a significant difference of the medians of the correlation distribution for all conserved miRNA-TargetScan target pairs compared to the correlation distribution of all remaining miRNA-mRNA pairs [14] (Figure 2). Considering that microarray mRNA expression data are less accurate in detecting poorly expressed transcripts, we investigated if the difference of the medians of the two correlation distributions (as quantified by the Wilcoxon-rank-sum-test) depended on a threshold of mRNA abundance (Figure 2, Additional file 2: Figure S1E-F). We set a threshold on mRNA abundance, selected the genes expressed above the threshold and computed the Pearson correlation between expression of miRNA families and their TargetScan targets. The difference of the medians of the two correlation distributions increased at a higher mRNA abundance threshold. To allow inclusion of a large number of mRNAs, we selected an mRNA abundance threshold of A >6.5 including 7,398 mRNAs (out of 16,783), resulting in a difference of 0.005 between the medians of the two correlation distributions (*P* value = 5.01e^-6^). For the TCGA dataset, using all 18,152 sequencing-detected mRNAs resulted in a difference between the medians of the two correlation distributions of 0.02 (*P* value = 6.8e^-120^), suggesting that an abundance threshold was not required (Additional file 2: Figure S2C-D). With the mRNA abundance thresholds defined above, higher expressed miRNAs overall demonstrated a more negative correlation with their respective TargetScan targets, having a larger effect on their targets and supported the previously selected threshold of RRF >1e^-4^ (Additional file 2: Figure S1G-H and S2E-F).

### Correlation analysis within molecular subtypes reveals varying degrees of miRNA target repression

Molecular subtypes with variability in expression of their dominant miRNAs, but less variability in their mRNA expression, are more likely to display negative miRNA-TargetScan-target correlations. Therefore, we conducted miRNA-mRNA correlation analyses by molecular subtypes of breast cancer [24] using the miRNA/mRNA abundance thresholds defined above. Our dataset [15] included 78 basal-like, 23 HER2, 25 luminal A, six luminal B, and 21 normal-like samples (10 carcinomas and 11 normal breast); eight samples could not be assigned to a particular subtype [25]. The 444 TCGA samples were subdivided to molecular subtypes using the PAM-50 classification scheme based on Agilent microarray data (84 basal, 52 HER2, 205 luminal A, 103 luminal B) [26].

**Figure 3:**
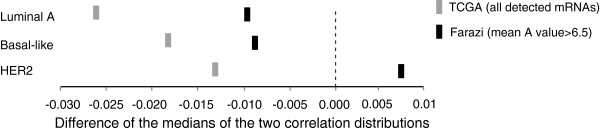
**Strength of negative miRNA-target correlations across molecular subtypes.** The difference of the medians of the distribution of conserved miRNA-TargetScan-target correlations compared to the distribution of the remainder miRNA-mRNA correlations for each molecular subtype. Results shown for [15], using an mRNA abundance threshold of mean A value >6.5, and [13], using all detected mRNAs.

Samples belonging to individual subtypes showed distinct differences of the medians of the correlation distributions comparing expression of miRNA-TargetScan-target pairs and all remaining miRNA-mRNA pairs: basal-like (-0.0088), luminal A (-0.0096), and normal-like (-0.011) (Wilcoxon-rank-sum test *P* value <0.05); the difference for the HER2 subtype (+0.0076) was not significant, even though it included a similar number of samples to the luminal A subtype (Figure 3). The TCGA dataset demonstrated similar results: the largest differences of median correlation values were noted for the basal-like (-0.018), luminal A (-0.026), and luminal B subtype (-0.017); the HER2 subtype displayed the smallest difference (-0.013) (*P* value <0.05) (Figure 3). Finally, we observed that different molecular subtypes displayed distinct correlations between expression of specific miRNA families and their respective top 10 anti-correlated conserved TargetScan-predicted targets among all samples, either in our or the TCGA dataset. For example, miR-17 family expression showed the strongest negative correlation with its targets within the basal-like subtype (Additional file 2: Figure S4). The rank of all miR-17 targets based on their anti-correlation with miR-17 expression between our dataset and the TCGA dataset showed fair concordance, with a Spearman correlation coefficient of 0.48 (*P* value <0.05) (Additional file 3: Table S2). To better quantify subtype-specific miRNA regulation, we rank miRNA-target associations within subtypes later in the manuscript.

### AGO2-PAR-CLIP-defined biochemical miRNA targets in MCF7 breast cancer cell line

To identify which miRNA-target pairs are more likely to display regulation, we used AGO2-PAR-CLIP [17] to capture biochemical miRNA targets and define their specific location within 3′ UTRs and CDSs, in the MCF7 luminal subtype and ER-positive/HER2-negative breast cancer ductal cell line [27]. Even though MCF7 cells display distinct mRNA profiles compared to cell lines belonging to the basal-like subtype (cell line subtypes defined in [27]), they share many abundant miRNAs with other breast cancer cell lines and tumors across all molecular subtypes [15]. MCF7 cells exhibit a drastic upregulation of miR-21, similar to breast tumors when compared to normal breast tissue [15].

**Figure 4:**
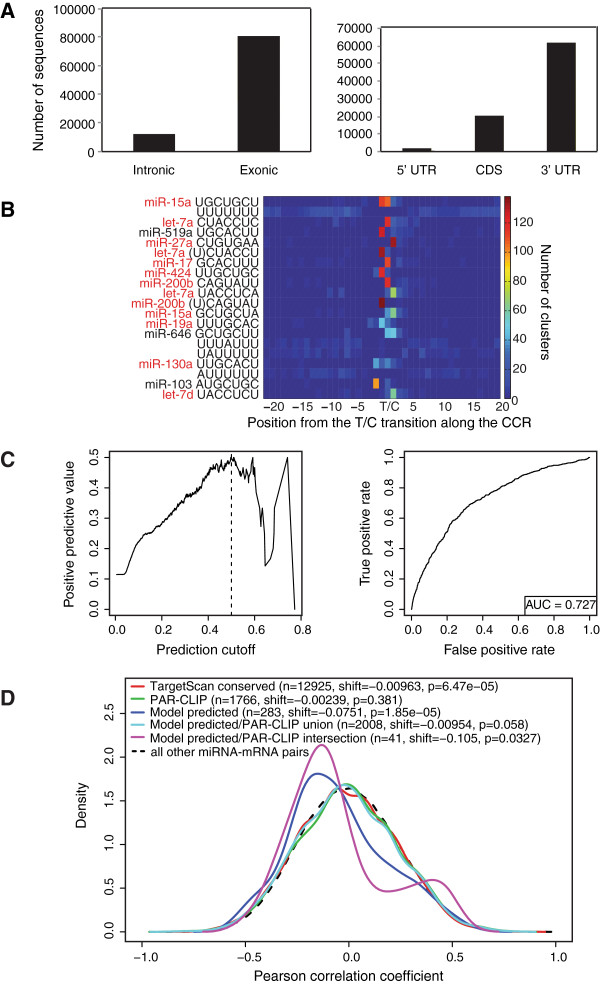
**AGO2-PAR-CLIP summary and regression model characteristics for the luminal A subtype[**[15]**].(A)** Genomic location of PAR-CLIP isolated mRNAs and distribution of AGO2 binding sites in transcript regions. Number of sequences included in clusters (clusters defined with ≥5 reads). **(B)** Representation of the 20 most significantly enriched 7-mer sequences within PAR-CLIP CCRs. T/C indicates the predominant T-to-C conversion defined by CCRs. **(C)** Regression model positive predictive value as a function of selected posterior probability score threshold on the left; AUC plot on the right. **(D)** Correlation density of expression of miRNA families and their conserved TargetScan, PAR-CLIP identified and model-predicted targets compared to the correlation density of all other miRNA and mRNA pairs.

We utilized a monoclonal anti-AGO2 antibody to isolate AGO2-associated RNAs [28,29]. Cells are grown in the presence of 4-thiouridine, which is incorporated into nascent RNA subsequently resulting in T-to-C conversion in cDNA reads recovered from crosslinked RNA to AGO2. The T-to-C conversion is a marker of selecting RNAs associated with AGO2 rather than background RNAs [17]. Our dataset demonstrated 80% and 40% T-to-C conversion for mRNA and miRNA reads, respectively, indicating the isolated RNAs were indeed crosslinked. The 341,490 mRNA-annotated sequences grouped into 4,879 clusters distributing across 2,539 transcripts (Additional file 4: Table S3A). The majority of reads (86.8%) were exonic, of which 73.6% were located in the 3′ UTR, 24.2% in the CDS and only 2% in the 5′ UTR (Figure 4A).

Crosslink-centered regions (CCRs) comprising 20 nucleotides (nt) upstream and downstream of the major T-to-C conversions within a cluster were generated to calculate all 16,384 possible 7-mers within the CCRs: the most significantly enriched 7-mers, relative to random sequences of the same dinucleotide composition corresponded to the reverse complement of the seed region (position 2-8) and other 7-mer combinations of abundant MCF7 miRNA families (let-7, miR-15a, miR-141, miR-17, miR-130a, miR-19a) (Table 1), consistent with previous observations in HEK293 cells [17]. Even though miR-21 was the most sequenced crosslinked miRNA, its complementary seed sequence was not identified among the top 20 7-mers. The enriched 7-mers were positioned 1-2 nt downstream of the predominant crosslinking site within the CCRs (Figure 4B), residing in the unpaired regions of the AGO protein ternary complex [30] as previously described [17]. We confirmed that enrichment of complementary 6- through 10-mer sequences to position 1-10 of the most abundant miRNAs was statistically significant within the isolated mRNAs compared to random sequences of the same di-nucleotide composition (Additional file 4: Table S3B-C) and produced a validated list of 7-mer m8 and 7-mer 1A miRNA target sites [31] (Additional file 4: Table S3D). This resulted in 3,597 canonical miRNA-target interactions, with some CCRs containing target sites for more than one miRNA. We focused on canonical miRNA binding sites, given that a previous study in our lab using AGO-PAR-CLIP in HEK293 cells [17] identified fewer than 6.6% non-canonical sites. Other recently described methodologies could be used to focus on non-canonical sites, but have not been directly compared to PAR-CLIP [32].

**Table 1:** Top expressed miRNA TargetScan families in MCF7 cells

**miRNA TargetScan family**	**RRF (%)**
miR-21	33.67
let-7	22.62
miR-17	4.28
miR-30a	3.06
miR-25	2.03
miR-19a	1.80
miR-27a	1.79
miR-23a	1.78
miR-26a	1.50
miR-7	1.50
miR-99a	1.50
miR-203	1.46
miR-378	1.32
miR-24	1.31
miR-141	1.30
miR-181a	1.27
miR-101	0.96
miR-22	0.88
miR-200b	0.84
miR-425	0.77
miR-15a	0.68
miR-375	0.67
miR-193	0.57
miR-374a	0.51
miR-320a	0.47
miR-182	0.44
miR-151-3p	0.43
miR-130a	0.42
miR-191	0.40
miR-148a	0.34
All other miRNAs	9.43

### Regression model predicts additional miRNA targets

TargetScan lists theoretically possible target sites within annotated 3′ UTRs, whereas PAR-CLIP provides evidence for expressed targets within MCF7 cells, and depending on sequencing depth may not have covered low-level-expressed miRNAs that may be more abundant in patient samples within different molecular subtypes. Using PAR-CLIP, we identified 3,597 canonical miRNA-target interactions (assuming seed sequence complementarity, including targets in the 3′ UTR and CDS), 2,584 of which were predicted by TargetScan (1,507 conserved and 1,077 non-conserved). To identify additional-subtype-specific miRNA targets from the large number of miRNA-TargetScan-target interactions (72,770 conserved and approximately 3.5 million non-conserved) and prioritize them, we followed a supervised machine learning approach (elastic net regression model; combination of LASSO and ridge regression). The goal of this approach was to build a model that can predict, based on characteristics of the miRNAs and their targets, whether a miRNA-target interaction is, in fact, a true interaction as determined by PAR-CLIP. As inputs to this model we used characteristics of the PAR-CLIP identified targets (number of 7-mer and 8-mer sites, conservation and context score derived from TargetScan) and their expression levels in patient subtypes (Additional file 5: Table S4 and Materials and methods for description). The training and test sets were constructed using all miRNA-TargetScan-target pairs that are: (1) expressed according to our miRNA and mRNA abundance thresholds in patients for each subtype; and (2) include an AGO2-crosslinked mRNA target (*n* = 10,200 for luminal A subtype). We used 5,106 for training the model and the remainder for testing model performance. As positive set we employed the crosslinked and PAR-CLIP-site seed-matched miRNA-TargetScan-target pairs (n = 561 for luminal A subtype). As negative set we employed crosslinked, but not PAR-CLIP-site seed-matched, miRNA-TargetScan-target pairs (n = 4,545) (Additional file 2: Figure S5). Our trained model allowed us to predict and rank miRNA-TargetScan-target pairs based on their likelihood of being ‘PAR-CLIP-like’ interactions (further details in Materials and methods).

For the luminal A subtype (which is the closest match to the MCF7 cell line in which the PAR-CLIP targets were determined), we obtained an area under the curve (AUC) of 0.73 for both training and test sets (Additional file 2: Figure S5). We chose a 0.5 threshold on the posterior probability, resulting in an FDR of approximately 0.5 (Figure 4C). We evaluated 12,925 conserved and 45,293 non-conserved miRNA-TargetScan-target interactions (meeting our miRNA and mRNA thresholds). We predicted 283 interactions from all TargetScan interactions, 41 of which were supported by PAR-CLIP, thus identifying 233 conserved and 9 non-conserved additional target interactions (additional 14%) [18]. These interactions involved 23 miRNA families, mainly let-7 and miR-29a. Model-predicted targets not identified by PAR-CLIP exhibited a median RPKM expression of 5 in MCF7 cells, compared to 14 for targets supported by PAR-CLIP (expression from [33]). This suggested that the regression model adds not only targets for highly expressed miRNAs in patient tissues (38 interactions including miR-125, miR-142-3p, miR-145, miR-199a, miR-21 and miR-34a), but also miRNA targets abundant in patient tissues missed from PAR-CLIP due to their lower abundance in MCF7 cells.

We observed a greater difference of the medians of the distribution of correlations for miRNA families and their model-predicted targets compared to the distribution of correlations of remaining miRNA-mRNA pairs, as opposed to miRNA-Targetscan targets and PAR-CLIP targets, supporting our approach (Figure 4D). The TCGA dataset showed similar results (Additional file 2: Figure S6).

We defined miRNA targets by taking the union of the biochemical PAR-CLIP and regression model-predicted targets calculated within each molecular subtype to focus on experimentally tractable targets. Irrespective of their behavior in patient data (inherent with variability due to sample annotation and profiling method, as well as feedback regulation) PAR-CLIP targets are supported by crosslinking evidence in a breast cancer cell line at a binding site resolution, while model-predicted targets resemble PAR-CLIP targets and result in a greater difference of the medians of the two correlation distributions. We will refer to this set of miRNA-target pairs as the Model Predicted and PAR-CLIP (MP-PCLIP) pairs (*n* = 2,008 in the luminal A subtype: 1,766 from PAR-CLIP and an additional 242 from model prediction).

To understand the contribution of each individual input to predict PAR-CLIP targets we conducted univariate correlation analyses (Additional file 5: Table S4). TargetScan total context score, aggregate conservation score, and number of conserved 7-mer and 8-mer sites showed the highest correlation to PAR-CLIP status, hence providing the most predictive power in the model [18,31,34]. We also observed that miRNA abundance in patient samples correlated with PAR-CLIP status, supporting a threshold in miRNA abundance required for measureable regulation of mRNAs.

### miRNA pathway associations across molecular subtypes

After selecting miRNA targets expressed in the different patient subtypes from the MP-PCLIP pairs, we used the Global Test (GT) to analyze miRNA-mRNA associations in the context of KEGG pathways [35]. The GT can be used to determine whether the global expression pattern of a group of gene sets is significantly related to a variable, as supported by either negative or positive correlations. We assessed whether miRNA expression significantly associated with expression of genes belonging to KEGG pathways (obtaining a GT *P* value for the association; results for each individual subtype and dataset can be obtained at [18]. The majority of miRNA-pathway associations that included MP-PCLIP targets, included a negative correlation between the miRNA and at least one of its respective targets. For the majority of miRNAs, miRNA-pathway associations that included an MP-PCLIP target showed lower *P* values compared to miRNA-pathway associations that did not (t-test *P* value <0.05), further validating our approach (Additional file 6: Table S5).

**Figure 5:**
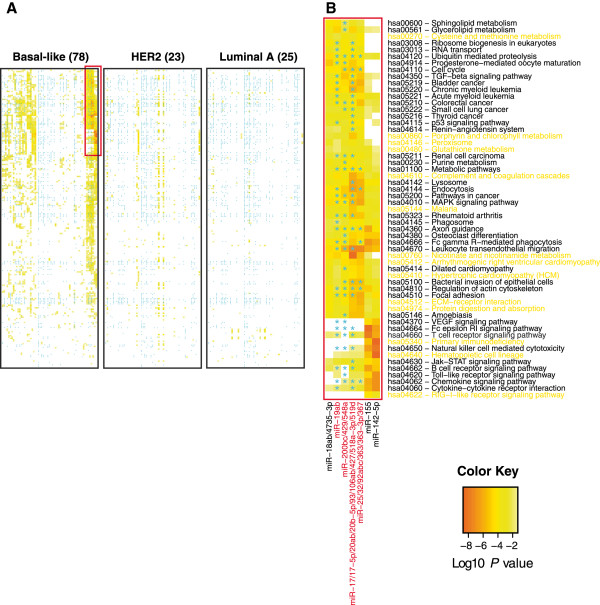
**miRNA-KEGG pathway associations.** Heatmaps depicting significant *P* values from GT correlating expression of miRNA families to genes belonging to KEGG pathways for different subtypes in [15]. Heatmaps for HER2 and luminal A subtype ordered according to the clustering of the basal-like subtype. Boxes labeled with stars illustrate presence of MP-PCLIP targets. Region selected by red outline represents area with highest concentration of significant *P* values seen in panel B. Color key depicts *P* values of associations. miRNAs in red include pathway gene associations with MP-PCLIP targets, while pathways in yellow do not.

For example, in the basal-like subtype, miRNA associated pathways included 1-469 expressed genes, of which 1-13 were MP-PCLIP targets, demonstrating negative or positive correlations to their regulating miRNA. Heatmaps of the GT association *P* values for each miRNA family expression with expression of genes belonging to each KEGG pathway, revealed different numbers of miRNA family-KEGG pathway associations in different molecular subtypes (Figure 5 and Additional file 2: Figure S7). The associations including an MP-PCLIP target are highlighted with a star. Moreover, pathways including miRNA-seed-matched PAR-CLIP targets illustrate activity in ductal cells.

As expected, most pathways were targeted by more than one miRNA. There was a large number of significant pathway associations for the miR-17, miR-19a, and miR-25 families in the basal-like subtype, with very few significant associations in the HER2 subtype in our dataset. The most significant miRNA-pathway association in the basal-like subtype was the association of miR-17 family with leukocyte transendothelial migration (*P* value = 3.5e^-8^), including a negative correlation between miR-17 family and its PAR-CLIP identified target CXCL12 [18] (Additional file 2: Figure S8). In the TCGA dataset, similarly to our dataset, miR-17 and miR-25 families showed many pathway associations within the basal-like subtype but not in the HER2 subtype.

### Ranking miRNA regulatory activity and tumor phenotype association across molecular subtypes

To elucidate miRNA-mediated regulation in the context of tumorigenesis, we performed an overall ranking of miRNAs by combining a number of evidence sources [36]. There are three components we considered in prioritizing miRNA regulatory activity: (1) association with its respective targets; (2) association with pathways - indicative of the miRNA’s ability to regulate its targets and in turn the pathways they regulate; and (3) association with cancer-related genes. A miRNA ranks high if achieving a high score (low *P* value) for each of the following statistical tests: (1) association of miRNA expression to expression of its respective targets based on the GT *P* value; (2) association of miRNA expression with expression of genes belonging to a KEGG pathway containing at least one MP-PCLIP target displaying either a negative or positive correlation with the miRNA (indicating functional relevance) (smallest GT *P* value out of all targeted pathways in KEGG); and (3) association of miRNA expression with expression of the gene set representing the Cancer Genome Census, modeling cancer relevance (GT *P* value) (see Materials and methods for further details). Each of the three tests is weighted equally in the ranking [36].

The top-scoring significant miRNA families of the overall ranking (using the significance test from [36]) in the basal-like subtype were miR-17, miR-19a, and miR-25 belonging to the oncogenic mir-17~92 cluster [37], and miR-200b, involved in the epithelial-mesenchymal transition [38] (Table 2) [18]. MiR-17 and miR-25 were also identified in the TCGA dataset. Expression of miR-17, miR-19a, and miR-200b targets was associated with distant metastasis-free survival in the basal-like subtype in a large cohort of breast cancer samples (see analysis in following section). Ranking of miRNA regulatory activity in the basal-like subtype showed fair concordance between our and the TCGA datasets, demonstrating a Spearman correlation coefficient of 0.47 (*P* value <0.05). MiR-24 was significant within the HER2 subtype, with miR-22 ranking second in our dataset (*P* value = 0.058). MiR-22 ranked second in the HER2 subtype in the TCGA dataset (*P* value = 0.215), but only reached statistical significance in the luminal B subtype (*P* value = 0).

**Table 2:** Top scoring miRNA TargetScan families in the Farazi and TCGA datasets

	**miRNA expression**			**miRNA activity**
	**Expression mean (RRF)**	**Expression min (RRF)**	**Expression max (RRF)**	**Rank test** ***P*** **value**
**Farazi 2011**				
**Basal-like (78)**				
miR-17	0.025	0.005	0.107	0.000
miR-200b	0.017	0.001	0.043	0.008
miR-25	0.009	0.002	0.041	0.008
miR-19a	0.024	0.001	0.111	0.049
**HER2 (23)**				
miR-24	0.019	0.005	0.058	0.017
miR-22	0.039	0.014	0.204	0.029
**Luminal A (25)**				
miR-425	0.003	4.38E-04	0.011	0.000
miR-101	0.011	0.001	0.038	0.000
miR-103a	0.025	0.005	0.099	0.002
**Luminal B (6)**				
miR-21	0.408	0.330	0.564	0.000
miR-33a	0.001	4.14E-04	0.002	0.022
**TCGA 2012**				
**Basal-like (84)**				
miR-17	0.016	0.003	0.056	0.012
miR-25	0.047	0.013	0.147	0.014
miR-142-3p	0.005	2.25E-04	0.024	0.025
**HER2 (52)**				
miR-142-3p	0.004	2.26E-04	0.018	0.004
miR-22	0.100	0.016	0.185	0.165^a^
**Luminal A (205)**				
miR-375	0.039	1.33E-04	0.228	0.079^a^
**Luminal B (103)**				
miR-22	0.084	0.023	0.213	0.000

Ranking of miRNA regulatory activity. Significant results shown for [15] and [13] (*P* value <0.05, unless otherwise noted^a^), in the context of miRNA family expression and variability.

At the same time, to elucidate miRNA tumor phenotype association in each subtype, we performed a second overall ranking of miRNAs by combining a set of evidence sources associated with patient histopathological and clinical characteristics, using the rank test described above [36]. These are GT *P* values assessing whether expression of miRNA families and their respective targets significantly related to development of distant metastasis and overall survival, number of positive lymph nodes, tumor size, lymphovascular invasion, and histological grade. The highest scoring miRNA family in our dataset was miR-130a in the basal-like subtype (Additional file 7: Table S6), regulating angiogenesis [39]. In the NKI295 dataset, which was used for validation of these results, miR-130a family ranked third, but did not reach statistical significance (Additional file 7: Table S6). Expression of miR-130a targets was also associated with distant metastasis-free survival and relapse-free survival in the basal-like subtype in a large cohort of breast cancer samples (see analysis in following section). Expression of miR-203 targets (implicated in cancer stem cell characteristics [40]) significantly correlated with lymphovascular invasion in the basal-like subtype in our dataset, a finding also supported in the luminal A subtype in the NKI295 dataset. It is interesting to note that the top ranked miRNAs according to regulatory activity do not necessarily overlap with the top ranked miRNAs according to association with tumor phenotype, but may be more interesting candidates for targeted therapy as they have a detectable regulatory role.

### Expression of miR-182 targets predicts metastasis

To determine whether expression levels of miRNAs and their respective targets predicted metastasis and overall survival, we used the GT with Cox-regression in our and the NKI295 study [3] (Additional file 7: Table S6). The NKI295 study includes mRNA microarray expression for 295 samples (55 luminal B, 123 luminal A, 29 normal-like, 53 basal-like, and 35 HER2). We selected 283 samples from patients with metastasis as first event to compare to our dataset. TCGA only reports overall survival with a short follow-up (average = 736 days), so we did not use it in this analysis. Expression of miR-182 targets, recently reported to be involved in breast cancer metastasis [41], was significantly associated with overall survival when considering all NKI295 patients. This prognostic signature comprised 12 genes with expression in the NKI295 series (XBP1, IGF1R, THBS1, PLAGL2, YWHAG, ZFP36, PSMC2, CCNG1, HSPA8, PFN1, ADCY6, NUP50). MiR-182 regulatory activity ranked fourth in the HER2 subtype in the TCGA dataset. None of the results within individual subtypes in our and the NKI295 dataset reached statistical significance after multiple testing correction and multivariate analysis accounting for histologic grade, tumor size, and lymph node status. However, we did notice weak concordance in the ranking of metastasis prognostic signatures between our and the NKI295 datasets in the basal-like and HER2 subtypes (correlation 0.35 and 0.43, *P* value <0.05). Finally, we further assessed the miRNA target prognostic signatures in two additional datasets (*n* = 623 (distant metastasis-free survival) and *n* = 1,616 (relapse-free survival)), using normalized mRNA expression from a large cohort of breast cancer samples [42,43]. The clinical and histopathological characteristics were unavailable, so we could not conduct multivariate analysis for these datasets. miR-183, which is co-expressed with miR-182, was the top prognostic signature in these datasets, with miR-182 still maintaining significance, providing some support for our results (Additional file 7: Table S6).

## Discussion

Functional studies in breast cancer cell lines and mouse models have suggested multiple roles played by miRNAs in the development of breast carcinomas and their metastatic potential involving targets regulating many cellular pathways. However, which miRNA-target pair(s) is (are) important in human disease progression is not always predicted by cell culture or animal model studies alone. Here we examined the extent of correlation in mRNA and miRNA expression in large sample collections by prioritizing the effects of miRNAs on many targets.

High miRNA abundance is critical for experimental analysis of transcriptome-wide seed-dependent target mRNA repression [44-47]. In our study we showed the importance of miRNA and mRNA abundance thresholds to focus on more reliably quantified and molecularly validated miRNA targets to conduct computational analysis of miRNA-mRNA correlations in tumor samples. The recent study by Dvinge et al. [14] did not impose sequence-based derived thresholds for miRNA expression and did not document miRNA repression in breast cancer, as suggested by lack of enrichment of negative correlations for miRNA-target pairs. Our approach documented miRNA and mRNA expression changes consistent with miRNA target regulation and focused on miRNA-target pairs based on their crosslinking to AGO2 through PAR-CLIP. This limited the large number of possible miRNA-TargetScan-target pairs to experimentally tractable pairs.

Even though miR-21 is highly expressed both in MCF7 cells and patient breast tumor samples, we were only able to identify a small number of its targets crosslinked by AGO2-PAR-CLIP. A recent article sheds some light into the targeting behavior of miR-21 [48]. They showed that miR-21 exhibited poor mRNA silencing activity in healthy mouse liver, despite being one of the top expressed miRNAs in this tissue, and suggested that reduced thermodynamic stability of seed pairing and target binding may contribute to this effect. At the same time, they were able to document target miR-21 regulation in HeLa cells, suggesting that the effect may be modulated by competition from AU-rich-RNA-binding proteins differentially expressed in distinct cell types.

We showed that conducting the analysis in each tumor subtype pointed to miRNAs and associated pathways that may represent therapeutic targets for specific groups of patients. Members of the mir-17~92 cluster had high miRNA regulatory activity (Table 2) in the basal-like subtype both in our and the TCGA dataset. MiR-17 and miR-19a families were associated with the leukocyte transendothelial migration pathway, with similarities to metastasis, and were negatively correlated with their PAR-CLIP target CXCL12. CXCL12, involved in metastasis [49], was also a PAR-CLIP target of other miRNA families (miR-7, miR-23a, miR-182, and miR-183) (Additional file 2: Figure S8).

Our prioritization of miRNA regulatory activity selects for miRNAs that show regulation through association with their respective targets and regulated pathways, as well as genes implicated in cancer, in distinct molecular subtypes. We consistently observed regulation by miRNAs in the basal-like subtype across two independent datasets. Detecting miRNA activity and cancer association does not necessarily predict whether inhibiting or over-expressing the miRNA will have therapeutic benefit - it simply points to the relevance of the prioritized miRNA as evidenced by repression of its targets in patient tissues. Two recent manuscripts also point to the importance of two of our top prioritized miRNA families: miR-200 and miR-22 [50,51] (Table 2). Song et al. found that miR-22 regulated breast cancer stemness and metastasis via TET-family-dependent chromatin remodeling. *In vitro* and *in vivo* experiments showed that miR-22 promoted epithelial mesenchymal transition and tumor invasion and metastasis. Our results point to high miR-22 activity in the luminal B subtype in the TCGA dataset, as well as the HER2 subtype in both datasets (ranked second with *P* value >0.05 in TCGA and *P* value <0.05 in our dataset). Another study by Pecot et al. showed that the miR-200 family blocked cancer angiogenesis specifically in the basal-like subtype. Our results point to high miR-200b family activity in the basal-like subtype in our dataset.

## Conclusions

Abundant miRNAs repress their respective targets in breast tumor-related processes, as documented by regulation of their targets in patient tissues. This regulation is subtle and may not be readily revealed in global analysis with a moderately large number of patient samples, but only by using approaches involving data curation and biochemical evidence, relying on miRNA sequencing-derived abundance. Moreover, this regulation may only be evident when conducting the analysis within individual molecular subtypes: for example, the extent of regulation as supported by pathway association in the HER2 subtype is less pronounced compared to the other subtypes.

We can only detect regulation for few highly abundant miRNAs, and can only validate three of these miRNAs across two independent datasets. Challenges and caveats to interpretation of our results include: (1) patient heterogeneity between the different patient datasets examined; (2) noise in the patient mRNA profiles due to the different platforms used for their detection (that is, sequencing *vs.* microarray); (3) assumptions made for the detection of miRNA targets, mainly focusing on targets that exhibit a negative correlation between their respective regulating miRNAs to derive thresholds for miRNA and mRNA abundance and negative or positive correlations for miRNA pathway association. Lack of detection of miRNA activity using our methodology does not necessarily rule out miRNA-mediated regulation; the analysis, instead, focuses on providing support from patient data for a few miRNAs that could be considered promising candidates for therapeutic manipulation. Finally, the challenges in validating prognostic signatures across datasets are not unique to our study, but represent frequent complexities arising from breast cancer heterogeneity and the different sets of genes detected by microarray and/or sequencing methodologies not allowing a direct comparison of gene expression signature performance.

In conclusion, we provide a list of miRNA targets, associated pathways, tumor phenotypes, and miRNA regulatory activity derived from patient samples as well as supported by biochemical evidence, to allow generation of clinically relevant hypotheses. Our analysis allows definition of a few specific miRNAs as potential therapeutic targets and prognostic markers in breast cancer and can be applied to other patient datasets.

## Materials and Methods

### Datasets and analysis

Our miRNA dataset was reported in [15]. mRNA abundance values (A) correspond to the fluorescence intensity averaged from both dye swap NKI Operon array experiments: defined as log_2_(sqrt(R*G)), where R and G are the red and green fluorescent channels. mRNA expression was normalized to a set of 100 tumors (log_2_(fold-change)). Probes correlating >0.8 were condensed to genes by averaging probe log_2_(fold-change). The TCGA dataset is described in [13] and was downloaded from ([52]; 2013-02). miRNA counts correspond to the most abundant isoform read measured for each miRNA within each sample and normalized to RRF. Detected miRNAs were defined as having more than 10 reads in at least 5% of the samples. Detected mRNAs were defined as having more than 20 reads in at least 5% of the samples. mRNA RPKM values of 0 were set to the lowest non-zero RPKM value measured in a given sample and subsequently log_2_-transformed. The NKI295 dataset is described in [3] and downloaded from [53], with an updated median follow-up of 12 years.

Intronic miRNAs were obtained from Table S2 in [54]. We excluded miRNAs with multiple copies, as they cannot be assigned to a single host gene. We used TargetScan version 6.2 [55] (context score and evolutionary conservation scores aggregated per gene and miRNA; Summary Counts file) and miRanda-miRSVR August 2010 release [56] (miRSVR scores aggregated per gene and miRNA). KEGG pathways were obtained from BioConductor [57], the CGC from [58] (Table_1_full_2012-01-18.xls). GT 5.12.0 and glmnet 1.9-3 packages were obtained from BioConductor version 2.11 (R version 2.15.3; 2013-03-01). Rank test for miRNA regulatory activity and phenotype association as described in [36]. Figure 1 and Additional file 2: Figure S9 describes the analysis outline and provides examples of the tables generated.

#### ***miRNA and mRNA abundance thresholds for patient data***

We evaluated thresholds for miRNA and mRNA expression to focus on higher confidence correlations. We established that overall expression of intronic miRNAs and their protein-coding host genes displayed a positive Pearson correlation, as described in [23,59] (Additional file 2: Figure S1A-B; Additional file 8: Table S7). We next investigated whether miRNA abundance influenced the positive correlations observed between expression of intronic miRNAs and their host genes. In our dataset, the correlation results for poorly expressed intronic miRNAs near the detection limit were more variable as compared to higher expressed miRNAs, which displayed stronger positive correlations with their host genes (*P* = 0.001) (Additional file 2: Figure S1C). mRNA abundance did not influence the correlation between intronic miRNAs and host genes, likely due to the non-linear variation in our array-based measurements (Additional file 2: Figure S1D). Hybridization-based mRNA arrays do not display the same linear variations for detection of lower expressed mRNAs, and may also reach saturation during detection of highly expressed mRNAs. We therefore set the miRNA expression threshold to an RRF of 1e^-4^ (corresponding to an average correlation of 0.28). Given that TCGA was sequenced deeper than our dataset (750,000 compared to 5,000 minimum reads per sample), almost all correlations between expression of intronic miRNAs and their host genes were positive (Additional file 2: Figure S2A).

#### ***TargetScan thresholds***

Applying more stringent TargetScan thresholds for aggregate conservation/PCT or total context score resulted in an even greater difference between the medians of the two correlation distributions at our selected miRNA and mRNA abundance thresholds (Additional file 2: Figure S10), further supporting the use of TargetScan.

#### ***Global tests***

We conducted the following GTs [35] for miRNA regulatory activity. First, we conducted a GT evaluating the association of miRNA expression with expression of its MP-PCLIP targets (miR ~ target1 + … + targetN). Second, we conducted a GT evaluating the association of miRNA expression with expression of gene sets corresponding to KEGG pathways (miR ~ kegg1.gene1 + … + kegg1.geneN,…, miR ~ keggK.gene1 + … + keggK.geneN) (examples can be found in Additional file 2: Figure S8). Third, we conducted a GT evaluating the association of miRNA expression with expression of the genes comprising the Cancer Gene Census (miR ~ cgc.gene1 + … + cgc.geneN). For tumor phenotype association, we conducted GTs evaluating the association of expression of a miRNA along with expression of its respective targets (miRNA target expression signature) to a particular tumor clinical or histopathological characteristic. We used logistic regression for association with lymph node status and lymphovascular invasion (yes or no), multinomial regression for tumor size (<2, 2-5, >5 cm) and histologic grade (good, moderate, poor), and Cox-regression for association with time to metastasis and overall survival (patient characteristics described in [15]). Multiple testing correction was conducted using the Benjamini-Hochberg method.

#### ***Regression model***

We used a combination of LASSO and ridge multivariate regression (glmnet package) to predict whether a given miRNA-TargetScan-target is a PAR-CLIP identified pair (true or false). As input to the model we employed the following variables: (A) TargetScan: aggregate conservation/PCT score, total context score, total number of conserved/non-conserved sites, total number of 7-mer m8, 7-mer 1A, and 8-mer conserved/non-conserved sites; (B) Patient data: miRNA/mRNA abundance/variance, miRNA-mRNA interaction terms (miRNA abundance multiplied by mRNA abundance considering sign of mRNA log_2_(fold-change), or irrespective of sign). We viewed the predictive model as hypothesis generating and not as a final set of high confidence pairs to have a larger set of miRNA-target pairs to include in further enrichment and association studies. Thus, we used a posterior probability prediction cutoff of 0.5 because it resulted in the best model performance, as judged by the positive predictive value (PPV) or FDR of 50%, yielding 283 miRNA-target pairs (Additional file 2: Figure S5). Increasing the posterior probability prediction cutoff to 0.7 for the TCGA dataset allowed us to reach an FDR of approximately 25%, but resulted in prediction of only 23 miRNA-target pairs (Additional file 2: Figure S6). Increasing the mRNA abundance threshold did not result in improvement in model performance (Additional file 2: Figure S11). Additional file 2: Figure S12 depicts the distribution of low- and high-expressed genes in the patient luminal A samples as a function of the MCF7 cell RPKM expression levels.

### Biochemical identification of miRNA targets using AGO2-PAR-CLIP

MCF7 cells were obtained from ATCC and grown at 37ºC in an atmosphere containing 5% CO_2_ in Dulbecco’s modified Eagle’s medium (1X D-MEM/high-glucose/L-glutamine/sodium pyruvate) supplemented with 10% heat inactivated fetal bovine serum, 100 unit/mL penicillin, 100 mg/mL streptomycin (Invitrogen, Sigma, and Gibco). Cells were grown in the presence of 100 μM 4-thiouridine (4SU) for 24 h and AGO2 complexes were immunoprecipitated using a monoclonal antibody against AGO2 (Millipore clone 9E8.2; used in [28,29]), according to [17]. We used lysis buffer in lieu of high-salt wash buffer to not disrupt the monoclonal antibody-bead interaction. Crosslinked RNA of 20-40 nt in length was recovered from the 100 kDa AGO2 immunoprecipitated protein complexes separated on SDS gel, confirmed by Western blot probing with a polyclonal antibody recognizing AGO2 (Millipore 07-590). The isolated RNA was converted into cDNA libraries, and sequenced by Illumina at the Rockefeller University Genomics Center. We analyzed the data similarly to [17]. The sequence reads were aligned to the human genome and transcript sequences from public databases, allowing for up to one mismatch. Overlapping reads >20 nt were clustered, and clusters containing <5 sequence reads or those with a content of <20% crosslinked sequences were not considered. A T-to-C conversation rate of 80% and 40% was noted for mRNA and miRNA reads, respectively. The lower T-to-C conversion rate for miRNAs was noted in our previous publication [17] and is likely due to the association of AGO2 with background abundant non-crosslinked miRNAs (such as, miR-21). miRNA targets were defined for the 69 top-expressed miRNAs in MCF7 cells (95% of miRNA sequence reads) by searching the sequences for complementary miRNA seed sequence sites (position 2-8, 1-7 perfect match, or allowing A at position 1), that are enriched within the isolated mRNAs compared to random sequences of the same di-nucleotide composition. The raw sequencing file is deposited with the Sequence Read Archive (SRX388831; [60]). Finally, we compared the number of conserved TargetScan and high miRSVR scoring Miranda miRNA-target interactions validated by PAR-CLIP. Accounting for expression of potential targets in MCF7 cells (RPKM >14), PAR-CLIP validated 8.3% of conserved TargetScan-predicted targets (3,104) and 9.9% of high miRSVR (<-1.2) scoring Miranda-predicted targets (1,970).

## Abbreviations

AGO2-PAR-CLIP: AGO2-Photoactivatable-ribonucleoside-enhanced crosslinking and immunoprecipitation; AUC: Area under the curve; CCR: Crosslink-centered region; CDS: Coding DNA sequence; DCIS: Ductal carcinoma in situ; FDR: False discovery rate; ESR/ER: Estrogen receptor; GT: Global test; IDC: Invasive ductal carcinoma; miRNA: MicroRNA; nt: Nucleotide; PGR/PR: Progesterone receptor; PPV: Positive predictive value; RPKM: Reads per kilobase per million; RRF: Relative read frequency; TCGA: The Cancer Genome Atlas; UTR: Untranslated region.

## Competing interests

TT is co-founder and scientific advisor to Alnylam Pharmaceuticals and scientific advisor to Regulus Therapeutics.

## Authors’ contributions

TAF conceived, designed, conducted experimental and computational work, and drafted the manuscript. JJH designed and conducted computational analysis, developed the interactive web-site (http://mp-pclip.nki.nl), and critically revised the manuscript. MB processed and analyzed the PAR-CLIP data. AM processed the PAR-CLIP experiment. HMH and MJV assisted with clinical and histopathological characteristics from patient datasets. TT and LFAW guided, critically revised the manuscript, and supervised the work. TT is an HHMI investigator and TAF is supported by the RUCCTS Grant #UL1RR024143. The work was supported in part by grant 1RC1CA145442. All authors read and approved the final manuscript.

## Supplementary Material

Additional file 1: Table S1 Summary of published studies. Click here for file

Additional file 2 Supplementary figures, figure legends and table legends. Click here for file

Additional file 3: Table S2 Correlation of ranking of miRNA targets between the Farazi 2011 and TCGA 2012 datasets for each individual miRNA within distinct molecular subtypes. Click here for file

Additional file 4: Table S3 AGO2-PAR-CLIP supplementary data. Click here for file

Additional file 5: Table S4 Regression model to predict miRNA ‘PAR-CLIP-like’ targets in patient datasets. Click here for file

Additional file 6: Table S5 T-test comparing *P* values of miRNA-pathway associations that include at least one MP-PCLIP miRNA target compared to *P* values of all other miRNA-pathway associations for the basal-like subtype in our dataset. Click here for file

Additional file 7: Table S6 Ranking lists for miRNA phenotype association and target prognostic signatures. Click here for file

Additional file 8: Table S7 Intronic miRNAs and their host genes whose expression shows positive correlation. Click here for file

## References

[B1] Curtis C Shah SP Chin SF Turashvili G Rueda OM Dunning MJ Speed D Lynch AG Samarajiwa S Yuan Y Graf S Ha G Haffari G Bashashati A Russell R McKinney S Langerod A Green A Provenzano E Wishart G Pinder S Watson P Markowetz F Murphy L Ellis I Purushotham A Borresen-Dale AL Brenton JD Tavare S Caldas C The genomic and transcriptomic architecture of 2,000 breast tumours reveals novel subgroups Nature 2012 486 346 352 22522925 10.1038/nature10983 PMC3440846

[B2] Wang Y Klijn JG Zhang Y Sieuwerts AM Look MP Yang F Talantov D Timmermans M Meijer-van Gelder ME Yu J Jatkoe T Berns EM Atkins D Foekens JA Gene-expression profiles to predict distant metastasis of lymph-node-negative primary breast cancer Lancet 2005 365 671 679 15721472 10.1016/S0140-6736(05)17947-1

[B3] van de Vijver MJ He YD van’t Veer LJ Dai H Hart AA Voskuil DW Schreiber GJ Peterse JL Roberts C Marton MJ Parrish M Atsma D Witteveen A Glas A Delahaye L van der Velde T Bartelink H Rodenhuis S Rutgers ET Friend SH Bernards R A gene-expression signature as a predictor of survival in breast cancer N Engl J Med 2002 347 1999 2009 10.1056/NEJMoa021967 12490681

[B4] Ventura A Jacks T MicroRNAs and cancer: short RNAs go a long way Cell 2009 136 586 591 10.1016/j.cell.2009.02.005 19239879 PMC3910108

[B5] Farazi TA Spitzer JI Morozov P Tuschl T miRNAs in human cancer J Pathol 2011 223 102 115 10.1002/path.2806 21125669 PMC3069496

[B6] Gurtan AM Sharp PA The role of miRNAs in regulating gene expression networks J Mol Biol 2013 425 3582 3600 10.1016/j.jmb.2013.03.007 23500488 PMC3757117

[B7] Blenkiron C Goldstein LD Thorne NP Spiteri I Chin SF Dunning MJ Barbosa-Morais NL Teschendorff AE Green AR Ellis IO Tavare S Caldas C EA M MicroRNA expression profiling of human breast cancer identifies new markers of tumor subtype Genome Biol 2007 8 R214 10.1186/gb-2007-8-10-r214 17922911 PMC2246288

[B8] Smeets A Daemen A Vanden Bempt I Gevaert O Claes B Wildiers H Drijkoningen R Van Hummelen P Lambrechts D De Moor B Neven P Sotiriou C Vandorpe T Paridaens R Christiaens MR Prediction of lymph node involvement in breast cancer from primary tumor tissue using gene expression profiling and miRNAs Breast Cancer Res Treat 2011 129 767 776 10.1007/s10549-010-1265-5 21116709

[B9] Enerly E Steinfeld I Kleivi K Leivonen SK Aure MR Russnes HG Ronneberg JA Johnsen H Navon R Rodland E Makela R Naume B Perala M Kallioniemi O Kristensen VN Yakhini Z Borresen-Dale AL miRNA-mRNA integrated analysis reveals roles for miRNAs in primary breast tumors PLoS One 2011 6 e16915 10.1371/journal.pone.0016915 21364938 PMC3043070

[B10] Van der Auwera I Limame R van Dam P Vermeulen PB Dirix LY Van Laere SJ Integrated miRNA and mRNA expression profiling of the inflammatory breast cancer subtype Br J Cancer 2010 103 532 541 10.1038/sj.bjc.6605787 20664596 PMC2939785

[B11] Buffa FM Camps C Winchester L Snell CE Gee HE Sheldon H Taylor M Harris AL Ragoussis J microRNA-associated progression pathways and potential therapeutic targets identified by integrated mRNA and microRNA expression profiling in breast cancer Cancer Res 2011 71 5635 5645 10.1158/0008-5472.CAN-11-0489 21737487

[B12] Cascione L Gasparini P Lovat F Carasi S Pulvirenti A Ferro A Alder H He G Vecchione A Croce CM Shapiro CL Huebner K Integrated MicroRNA and mRNA signatures associated with survival in triple negative breast cancer PLoS One 2013 8 e55910 10.1371/journal.pone.0055910 23405235 PMC3566108

[B13] TCGA Comprehensive molecular portraits of human breast tumours Nature 2012 490 61 70 10.1038/nature11412 23000897 PMC3465532

[B14] Dvinge H Git A Graf S Salmon-Divon M Curtis C Sottoriva A Zhao Y Hirst M Armisen J Miska EA Chin SF Provenzano E Turashvili G Green A Ellis I Aparicio S Caldas C The shaping and functional consequences of the microRNA landscape in breast cancer Nature 2013 497 378 382 10.1038/nature12108 23644459

[B15] Farazi TA Horlings HM Ten Hoeve JJ Mihailovic A Halfwerk H Morozov P Brown M Hafner M Reyal F van Kouwenhove M Kreike B Sie D Hovestadt V Wessels LF van de Vijver MJ Tuschl T MicroRNA sequence and expression analysis in breast tumors by deep sequencing Cancer Res 2011 71 4443 4453 10.1158/0008-5472.CAN-11-0608 21586611 PMC3129492

[B16] Lewis BP Burge CB Bartel DP Conserved seed pairing, often flanked by adenosines, indicates that thousands of human genes are microRNA targets Cell 2005 120 15 20 10.1016/j.cell.2004.12.035 15652477

[B17] Hafner M Landthaler M Burger L Khorshid M Hausser J Berninger P Rothballer A Ascano M Jr Jungkamp AC Munschauer M Ulrich A Wardle GS Dewell S Zavolan M Tuschl T Transcriptome-wide identification of RNA-binding protein and microRNA target sites by PAR-CLIP Cell 2010 141 129 141 10.1016/j.cell.2010.03.009 20371350 PMC2861495

[B18] MicroRNA target explorer for breast cancer data [http://mp-pclip.nki.nl]

[B19] Bartel DP MicroRNAs: target recognition and regulatory functions Cell 2009 136 215 233 10.1016/j.cell.2009.01.002 19167326 PMC3794896

[B20] Rajewsky N microRNA target predictions in animals Nat Genet 2006 Suppl S8 S13 16736023 10.1038/ng1798

[B21] Alexiou P Maragkakis M Papadopoulos GL Reczko M Hatzigeorgiou AG Lost in translation: an assessment and perspective for computational microRNA target identification Bioinformatics 2009 25 3049 3055 10.1093/bioinformatics/btp565 19789267

[B22] Betel D Wilson M Gabow A Marks DS Sander C The microRNA.org resource: targets and expression Nucleic Acids Res 2008 36 D149 D153 18158296 10.1093/nar/gkm995 PMC2238905

[B23] Creighton CJ Hernandez-Herrera A Jacobsen A Levine DA Mankoo P Schultz N Du Y Zhang Y Larsson E Sheridan R Xiao W Spellman PT Getz G Wheeler DA Perou CM Gibbs RA Sander C Hayes DN Gunaratne PH Integrated analyses of microRNAs demonstrate their widespread influence on gene expression in high-grade serous ovarian carcinoma PLoS One 2012 7 e34546 10.1371/journal.pone.0034546 22479643 PMC3315571

[B24] Perou CM Sorlie T Eisen MB van de Rijn M Jeffrey SS Rees CA Pollack JR Ross DT Johnsen H Akslen LA Fluge O Pergamenschikov A Williams C Zhu SX Lonning PE Borresen-Dale AL Brown PO Botstein D Molecular portraits of human breast tumours Nature 2000 406 747 752 10.1038/35021093 10963602

[B25] Hu Z Fan C Oh DS Marron JS He X Qaqish BF Livasy C Carey LA Reynolds E Dressler L Nobel A Parker J Ewend MG Sawyer LR Wu J Liu Y Nanda R Tretiakova M Ruiz Orrico A Dreher D Palazzo JP Perreard L Nelson E Mone M Hansen H Mullins M Quackenbush JF Ellis MJ Olopade OI Bernard PS **The molecular portraits of breast tumors are conserved across microarray platforms** BMC Genomics 2006 7 96 10.1186/1471-2164-7-96 16643655 PMC1468408

[B26] Cerami E Gao J Dogrusoz U Gross BE Sumer SO Aksoy BA Jacobsen A Byrne CJ Heuer ML Larsson E Antipin Y Reva B Goldberg AP Sander C Schultz N The cBio cancer genomics portal: an open platform for exploring multidimensional cancer genomics data Cancer Discov 2012 2 401 404 10.1158/2159-8290.CD-12-0095 22588877 PMC3956037

[B27] Kao J Salari K Bocanegra M Choi YL Girard L Gandhi J Kwei KA Hernandez-Boussard T Wang P Gazdar AF Minna JD Pollack JR **Molecular profiling of breast cancer cell lines defines relevant tumor models and provides a resource for cancer gene discovery** PLoS One 2009 4 e6146 10.1371/journal.pone.0006146 19582160 PMC2702084

[B28] Lipchina I Elkabetz Y Hafner M Sheridan R Mihailovic A Tuschl T Sander C Studer L Betel D Genome-wide identification of microRNA targets in human ES cells reveals a role for miR-302 in modulating BMP response Genes Dev 2011 25 2173 2186 10.1101/gad.17221311 22012620 PMC3205587

[B29] Skalsky RL Corcoran DL Gottwein E Frank CL Kang D Hafner M Nusbaum JD Feederle R Delecluse HJ Luftig MA Tuschl T Ohler U Cullen BR The viral and cellular microRNA targetome in lymphoblastoid cell lines PLoS Pathog 2012 8 e1002484 10.1371/journal.ppat.1002484 22291592 PMC3266933

[B30] Wang Y Juranek S Li H Sheng G Tuschl T Patel DJ Structure of an argonaute silencing complex with a seed-containing guide DNA and target RNA duplex Nature 2008 456 921 926 10.1038/nature07666 19092929 PMC2765400

[B31] Friedman RC Farh KK Burge CB Bartel DP Most mammalian mRNAs are conserved targets of microRNAs Genome Res 2009 19 92 105 18955434 10.1101/gr.082701.108 PMC2612969

[B32] Helwak A Kudla G Dudnakova T Tollervey D Mapping the human miRNA interactome by CLASH reveals frequent noncanonical binding Cell 2013 153 654 665 10.1016/j.cell.2013.03.043 23622248 PMC3650559

[B33] Wang ET Sandberg R Luo S Khrebtukova I Zhang L Mayr C Kingsmore SF Schroth GP Burge CB Alternative isoform regulation in human tissue transcriptomes Nature 2008 456 470 476 10.1038/nature07509 18978772 PMC2593745

[B34] Grimson A Farh KK Johnston WK Garrett-Engele P Lim LP Bartel DP MicroRNA targeting specificity in mammals: determinants beyond seed pairing Mol Cell 2007 27 91 105 10.1016/j.molcel.2007.06.017 17612493 PMC3800283

[B35] Goeman JJ van de Geer SA de Kort F van Houwelingen HC A global test for groups of genes: testing association with a clinical outcome Bioinformatics 2004 20 93 99 10.1093/bioinformatics/btg382 14693814

[B36] Aerts S Lambrechts D Maity S Van Loo P Coessens B De Smet F Tranchevent LC De Moor B Marynen P Hassan B Carmeliet P Moreau Y Gene prioritization through genomic data fusion Nat Biotechnol 2006 24 537 544 10.1038/nbt1203 16680138

[B37] Olive V Jiang I He L mir-17-92, a cluster of miRNAs in the midst of the cancer network Int J Biochem Cell Biol 2010 42 1348 1354 10.1016/j.biocel.2010.03.004 20227518 PMC3681296

[B38] Brabletz S Brabletz T The ZEB/miR-200 feedback loop–a motor of cellular plasticity in development and cancer? EMBO Rep 2010 11 670 677 10.1038/embor.2010.117 20706219 PMC2933868

[B39] Chen Y Gorski DH Regulation of angiogenesis through a microRNA (miR-130a) that down-regulates antiangiogenic homeobox genes GAX and HOXA5 Blood 2008 111 1217 1226 17957028 10.1182/blood-2007-07-104133 PMC2214763

[B40] DeCastro AJ Dunphy KA Hutchinson J Balboni AL Cherukuri P Jerry DJ DiRenzo J MiR203 mediates subversion of stem cell properties during mammary epithelial differentiation via repression of DeltaNP63alpha and promotes mesenchymal-to-epithelial transition Cell Death Dis 2013 4 e514 10.1038/cddis.2013.37 23449450 PMC3734833

[B41] Lei R Tang J Zhuang X Deng R Li G Yu J Liang Y Xiao J Wang HY Yang Q Hu G Suppression of MIM by microRNA-182 activates RhoA and promotes breast cancer metastasis Oncogene 2013 doi:10.1038/onc.2013.65 10.1038/onc.2013.65 23474751

[B42] Gyorffy B Schafer R Meta-analysis of gene expression profiles related to relapse-free survival in 1,079 breast cancer patients Breast Cancer Res Treat 2009 118 433 441 10.1007/s10549-008-0242-8 19052860

[B43] Staiger C Cadot S Gyorffy B Wessels LF Klau W Current composite-feature classification methods do not outperform simple single-genes classifiers in breast cancer prognosis Frontiers in Genetics 2013 4 289 24391662 10.3389/fgene.2013.00289 PMC3870302

[B44] Krutzfeldt J Rajewsky N Braich R Rajeev KG Tuschl T Manoharan M Stoffel M Silencing of microRNAs in vivo with 'antagomirs' Nature 2005 438 685 689 10.1038/nature04303 16258535

[B45] Baek D Villen J Shin C Camargo FD Gygi SP Bartel DP The impact of microRNAs on protein output Nature 2008 455 64 71 10.1038/nature07242 18668037 PMC2745094

[B46] Selbach M Schwanhausser B Thierfelder N Fang Z Khanin R Rajewsky N Widespread changes in protein synthesis induced by microRNAs Nature 2008 455 58 63 10.1038/nature07228 18668040

[B47] Linsley PS Schelter J Burchard J Kibukawa M Martin MM Bartz SR Johnson JM Cummins JM Raymond CK Dai H Chau N Cleary M Jackson AL Carleton M Lim L Transcripts targeted by the microRNA-16 family cooperatively regulate cell cycle progression Mol Cell Biol 2007 27 2240 2252 10.1128/MCB.02005-06 17242205 PMC1820501

[B48] Androsavich JR Chau BN Bhat B Linsley PS Walter NG Disease-linked microRNA-21 exhibits drastically reduced mRNA binding and silencing activity in healthy mouse liver RNA 2012 18 1510 1526 10.1261/rna.033308.112 22740638 PMC3404372

[B49] Wendt MK Cooper AN Dwinell MB Epigenetic silencing of CXCL12 increases the metastatic potential of mammary carcinoma cells Oncogene 2008 27 1461 1471 10.1038/sj.onc.1210751 17724466

[B50] Pecot CV Rupaimoole R Yang D Akbani R Ivan C Lu C Wu S Han HD Shah MY Rodriguez-Aguayo C Bottsford-Miller J Liu Y Kim SB Unruh A Gonzalez-Villasana V Huang L Zand B Moreno-Smith M Mangala LS Taylor M Dalton HJ Sehgal V Wen Y Kang Y Baggerly KA Lee JS Ram PT Ravoori MK Kundra V Zhang X Tumour angiogenesis regulation by the miR-200 family Nat Commun 2013 4 2427 24018975 10.1038/ncomms3427 PMC3904438

[B51] Song SJ Ito K Ala U Kats L Webster K Sun SM Jongen-Lavrencic M Manova-Todorova K Teruya-Feldstein J Avigan DE Delwel R Pandolfi PP The oncogenic microRNA miR-22 targets the TET2 tumor suppressor to promote hematopoietic stem cell self-renewal and transformation Cell Stem Cell 2013 13 87 101 10.1016/j.stem.2013.06.003 23827711 PMC3767186

[B52] BioPortal for Cancer Genomics [http://www.cbioportal.org/public-portal/]

[B53] Computational Cancer Biology: Division of Molecular Carcinogenesis, Netherlands Cancer Institute [http://ccb.nki.nl/data.php]

[B54] Radfar M Wong W Morris QD Predicting the target genes of intronic microRNAs using large-scale gene expression data Conf Proc IEEE Eng Med Biol Soc 2010 2010 791 794 21096111 10.1109/IEMBS.2010.5626505

[B55] TargetScan Human: Prediction of microRNA targets [http://www.targetscan.org]

[B56] microRNA.org – Targets and Expession [http://microRNA.org]

[B57] [ftp://ftp.genome.jp/pub/kegg/pathway 2011-03-14]

[B58] COSMIC: Catalogue of somatic mutations in cancer [http://www.sanger.ac.uk/genetics/CGP/Census/]

[B59] Baskerville S Bartel DP Microarray profiling of microRNAs reveals frequent coexpression with neighboring miRNAs and host genes RNA 2005 11 241 247 10.1261/rna.7240905 15701730 PMC1370713

[B60] The Sequence Read Archive (SRA) http://www.ncbi.nlm.nih.gov/sra/?term=SRX388831 10.1093/nar/gkq1019 PMC3013647 21062823

