# Supplementary material for: Identification of distinct miRNA target regulation between breast cancer molecular subtypes using AGO2-PAR-CLIP and patient datasets
Source: Genome Biol. 2014 Jan 7;15(1):R9. doi: 10.1186/gb-2014-15-1-r9 (PMC4053773; doi:10.1186/gb-2014-15-1-r9)
Supplement: Additional file 2 — Supplementary figures, figure legends and table legends. [file gb-2014-15-1-r9-S2.pdf]

Supplementary Figure 1

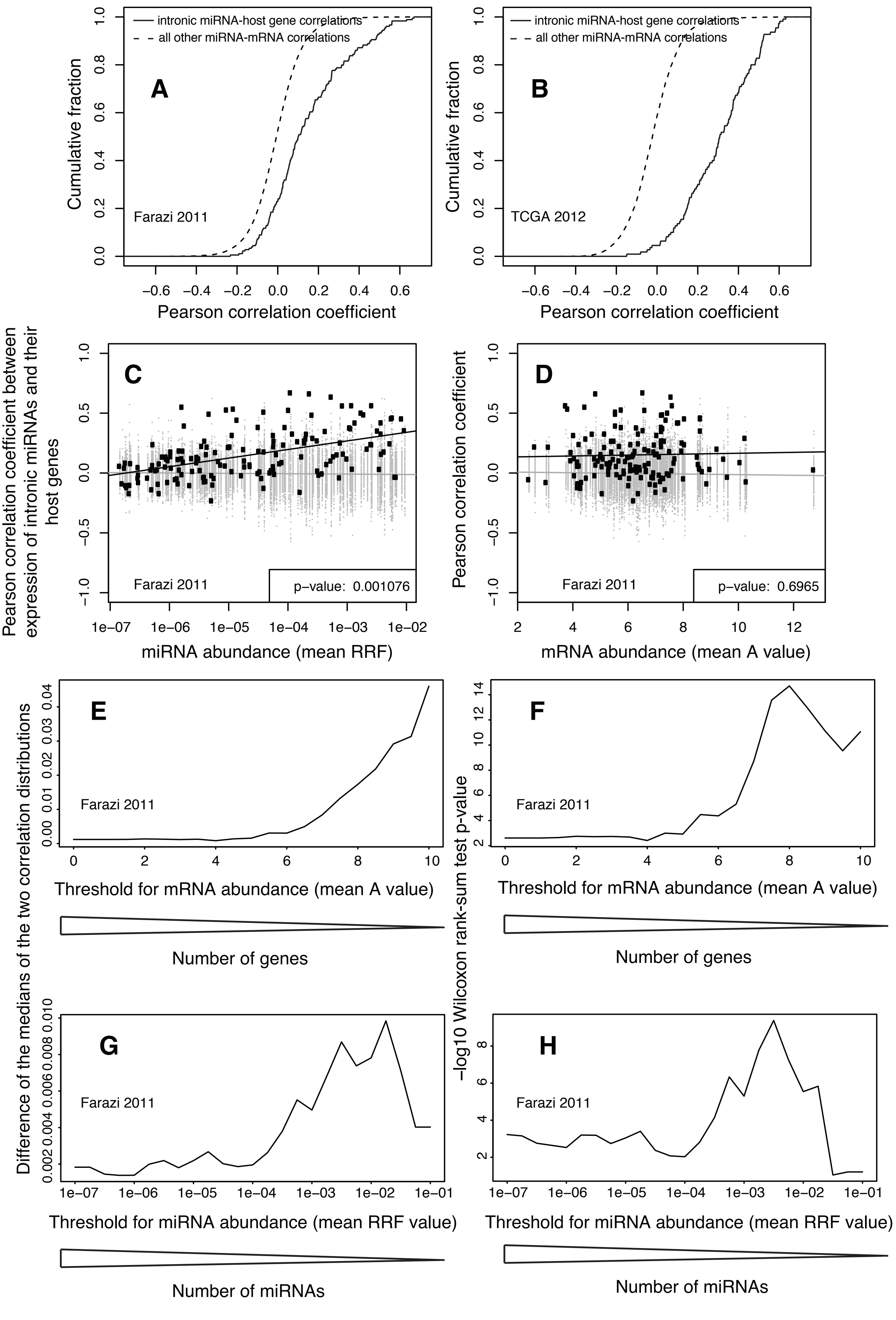

Supplementary Figure 1 **Farazi 2011**

I

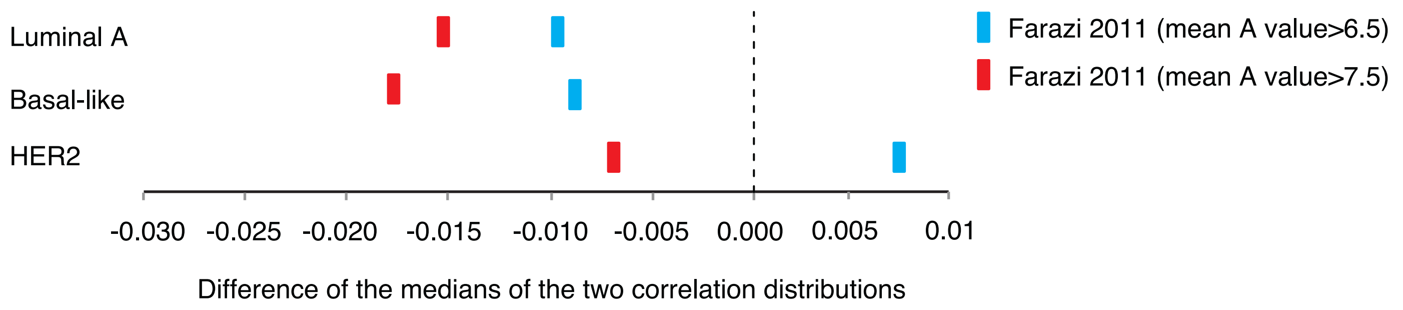

Supplementary Figure 2 **TCGA 2012**

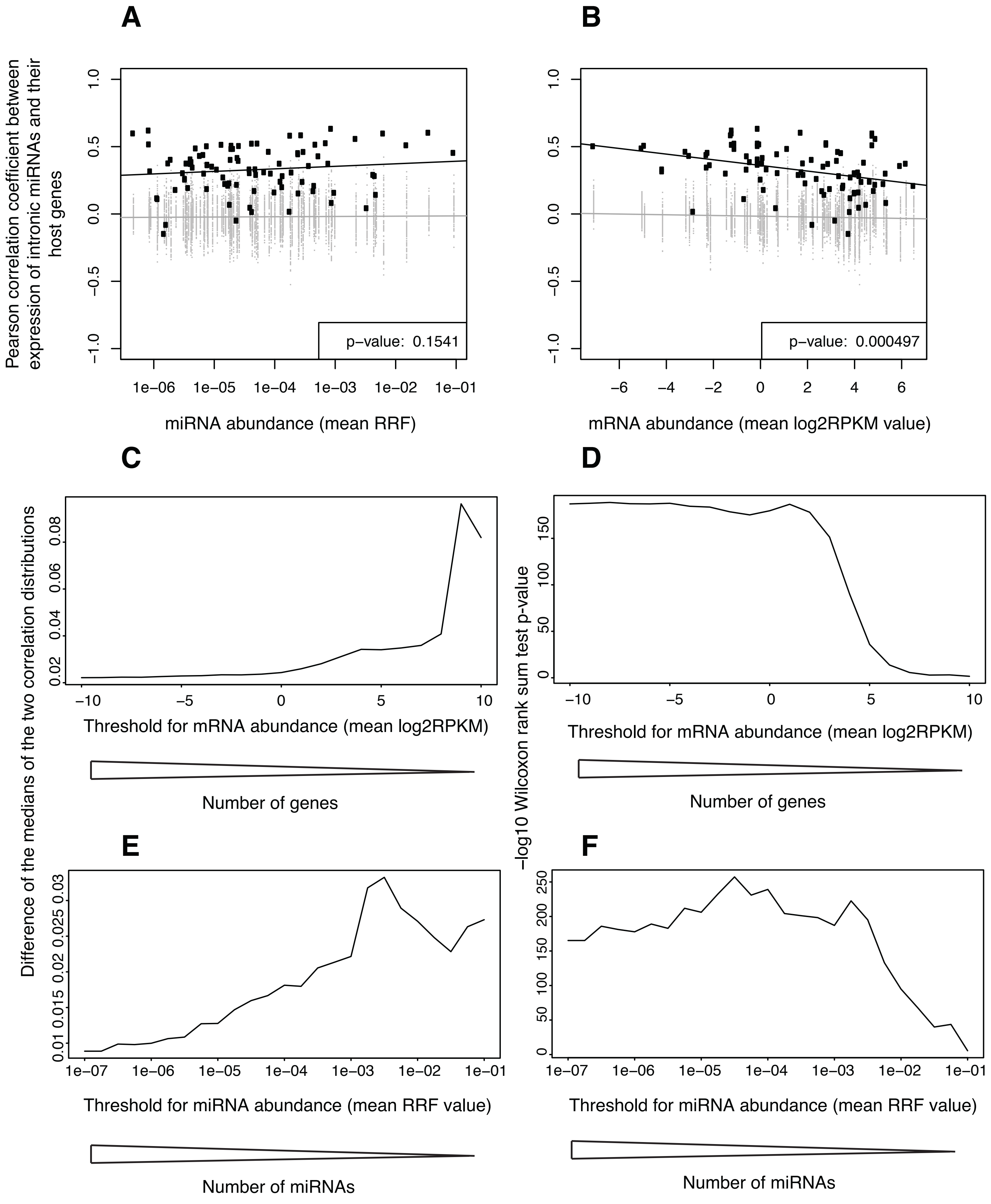

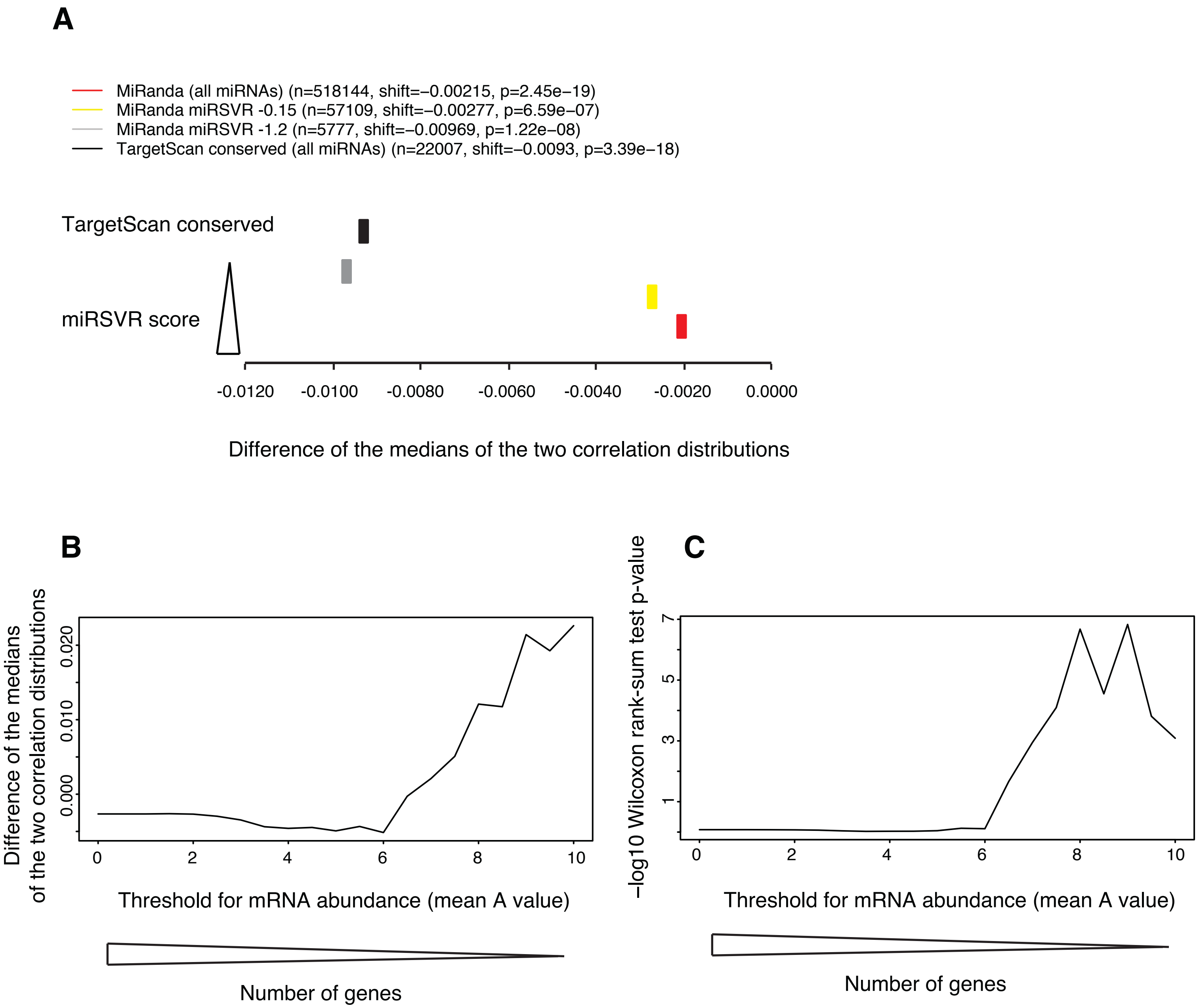
A
MiRanda (all miRNAs) (n=518144, shift=−0.00215, p=2.45e−19)
MiRanda miRSVR -0.15 (n=57109, shift=−0.00277, p=6.59e−07)
MiRanda miRSVR -1.2 (n=5777, shift=−0.00969, p=1.22e−08)
TargetScan conserved (all miRNAs) (n=22007, shift=−0.0093, p=3.39e−18)
TargetScan conserved
miRSVR score
-0.0120
-0.0100
-0.0080
-0.0060
-0.0040
-0.0020
0.0000
Difference of the medians of the two correlation distributions
B
Difference of the medians of the two correlation distributions
0.000
0.010
0.020
0
2
4
6
8
10
Threshold for mRNA abundance (mean A value)
Number of genes
C
−log10 Wilcoxon rank-sum test p-value
1
3
5
7
0
2
4
6
8
10
Threshold for mRNA abundance (mean A value)
Number of genes

Supplementary Figure 4

A **MMP2, MGLL, RHOC, CDKN1A, NBL1, WFS1, ARHGAP1, MAP3K12, FBXL5, TRIOBP (selection based on Farazi 2011)**

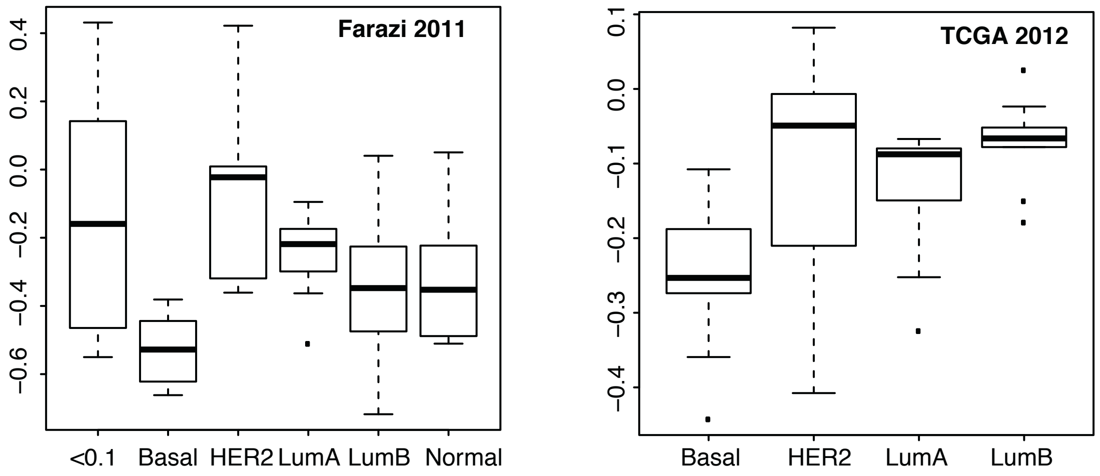

B **LIMA1, PRR15, TBC1D9, FOXA1, ZBTB4, FBXL5, CYBRD1, SLC40A1, NTN4, KIAA0247 (selection based on TCGA 2012)**

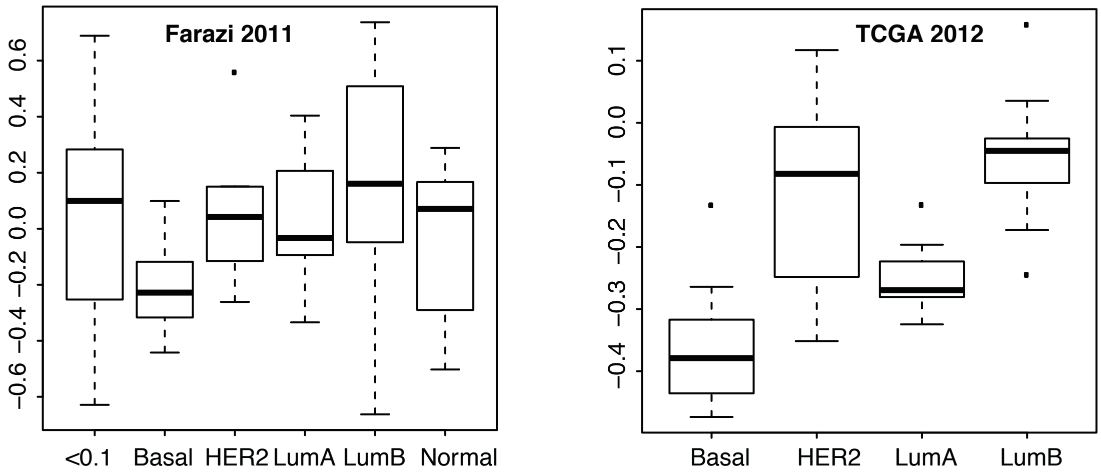

Supplementary Figure 5

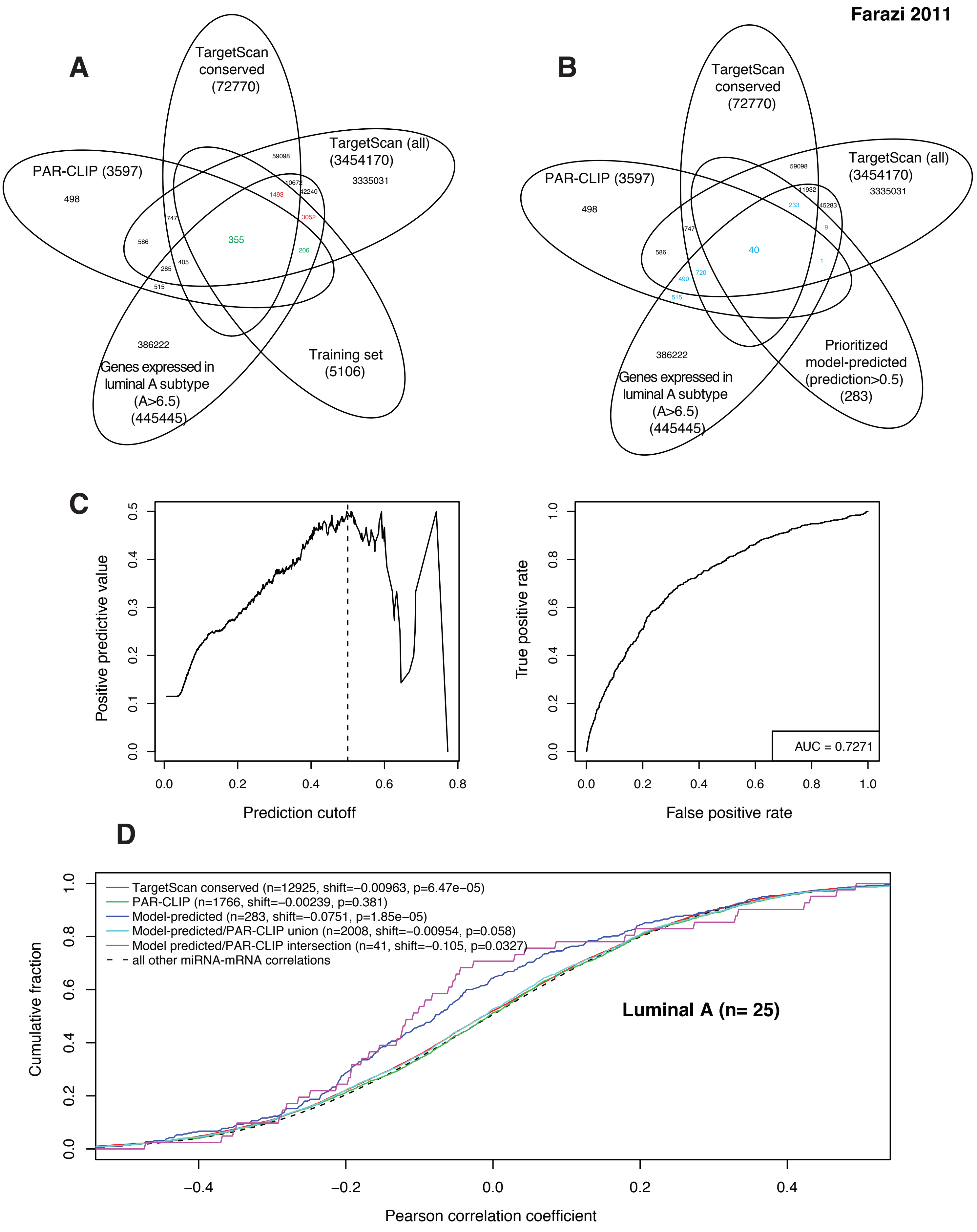

Supplementary Figure 5

Farazi 2011

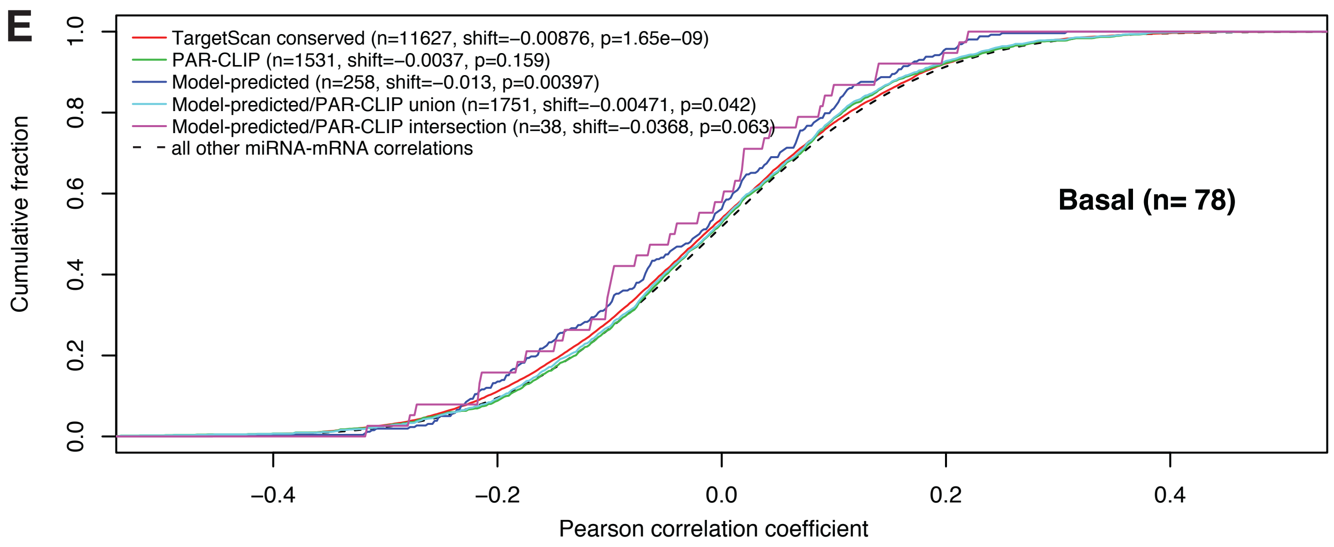

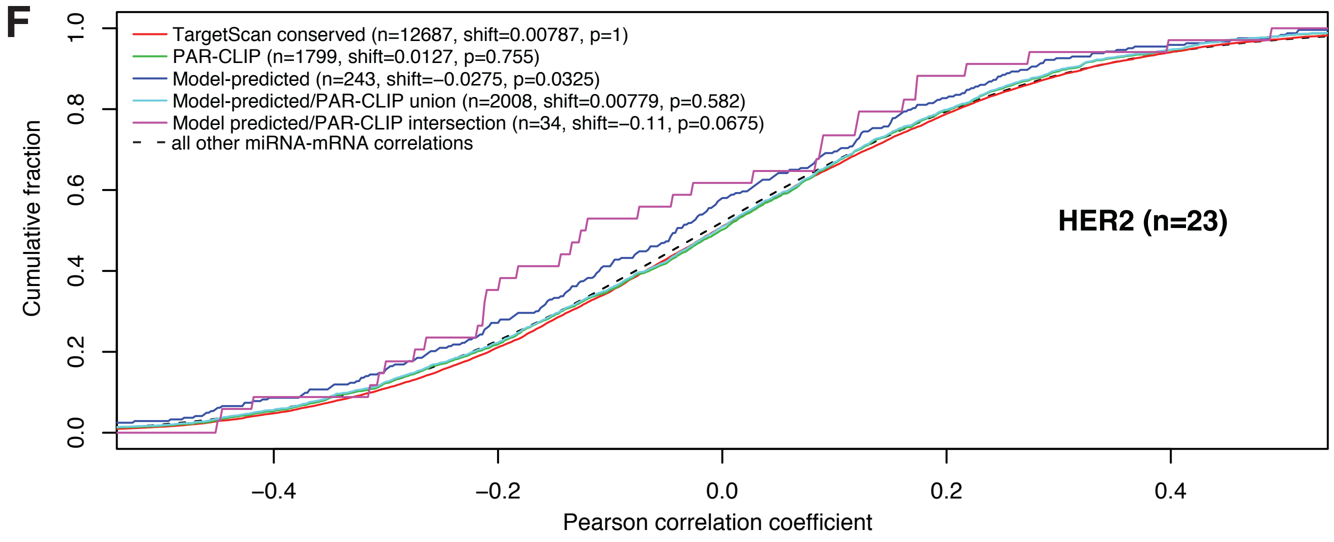

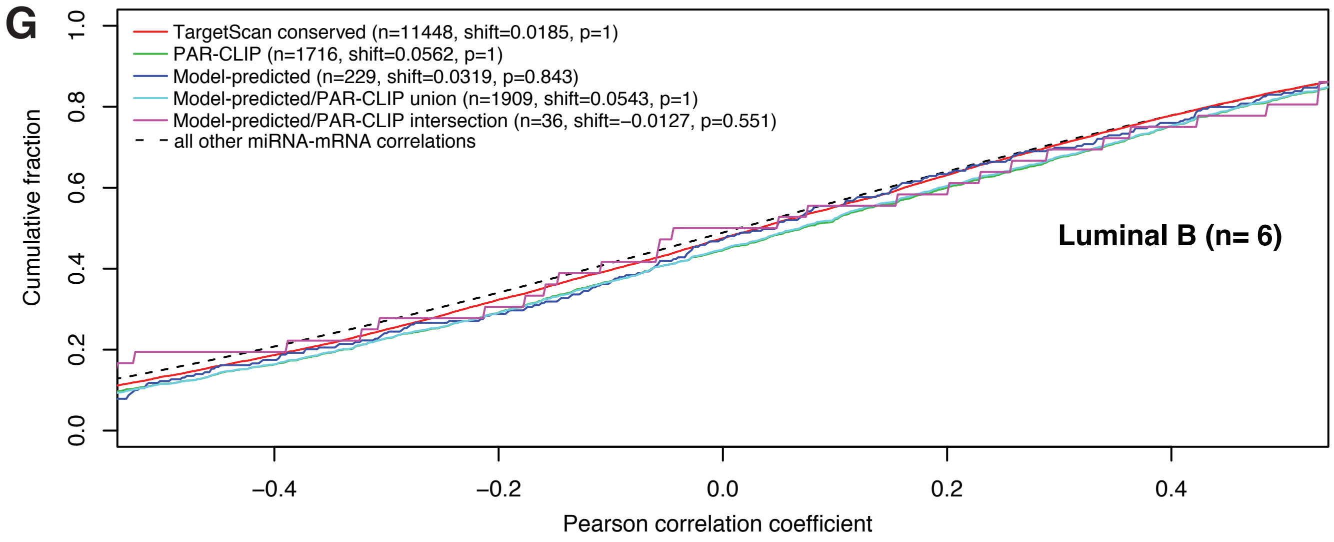

Supplementary Figure 6

TCGA 2012

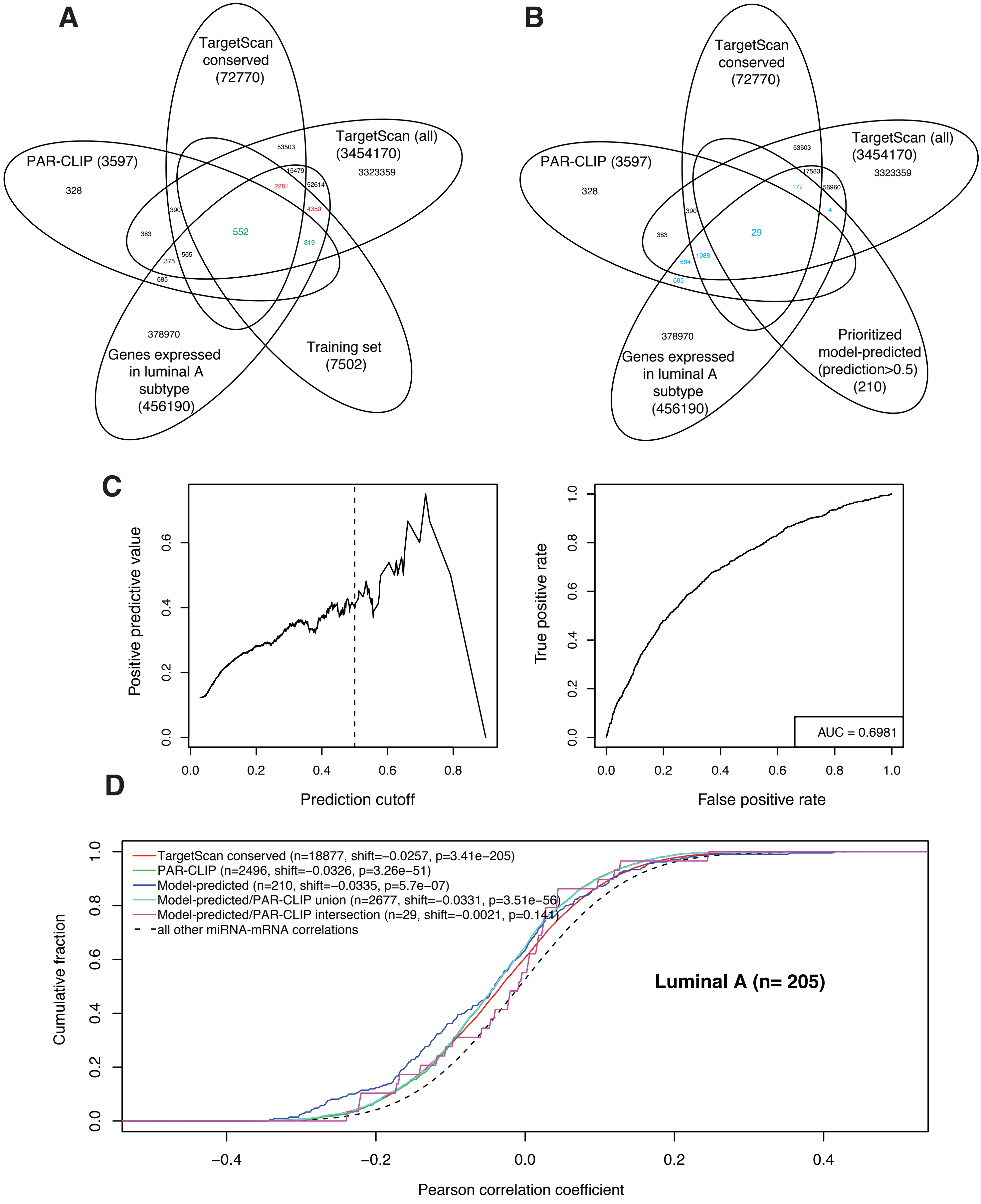

Supplementary Figure 6

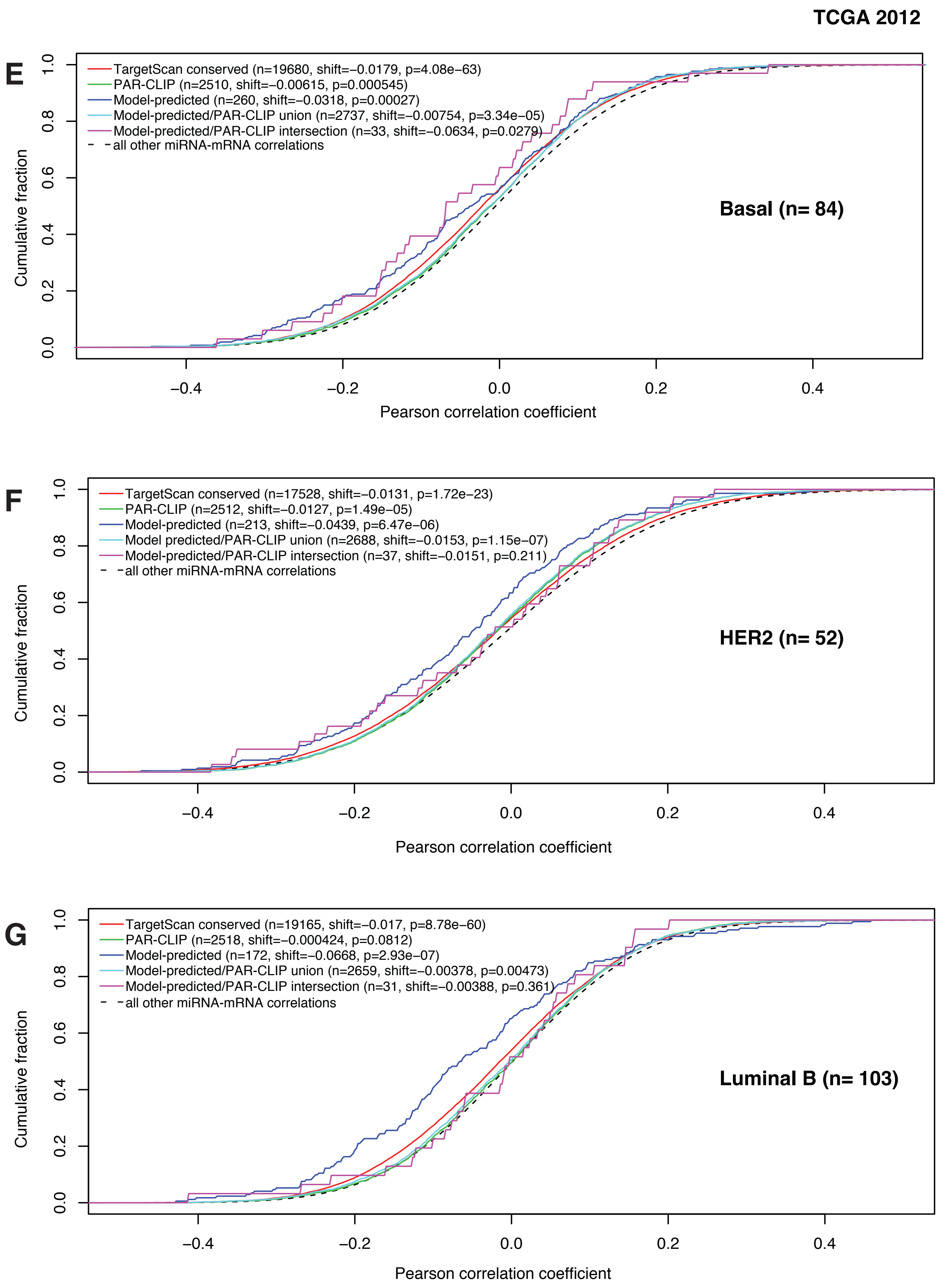

Supplementary Figure 7

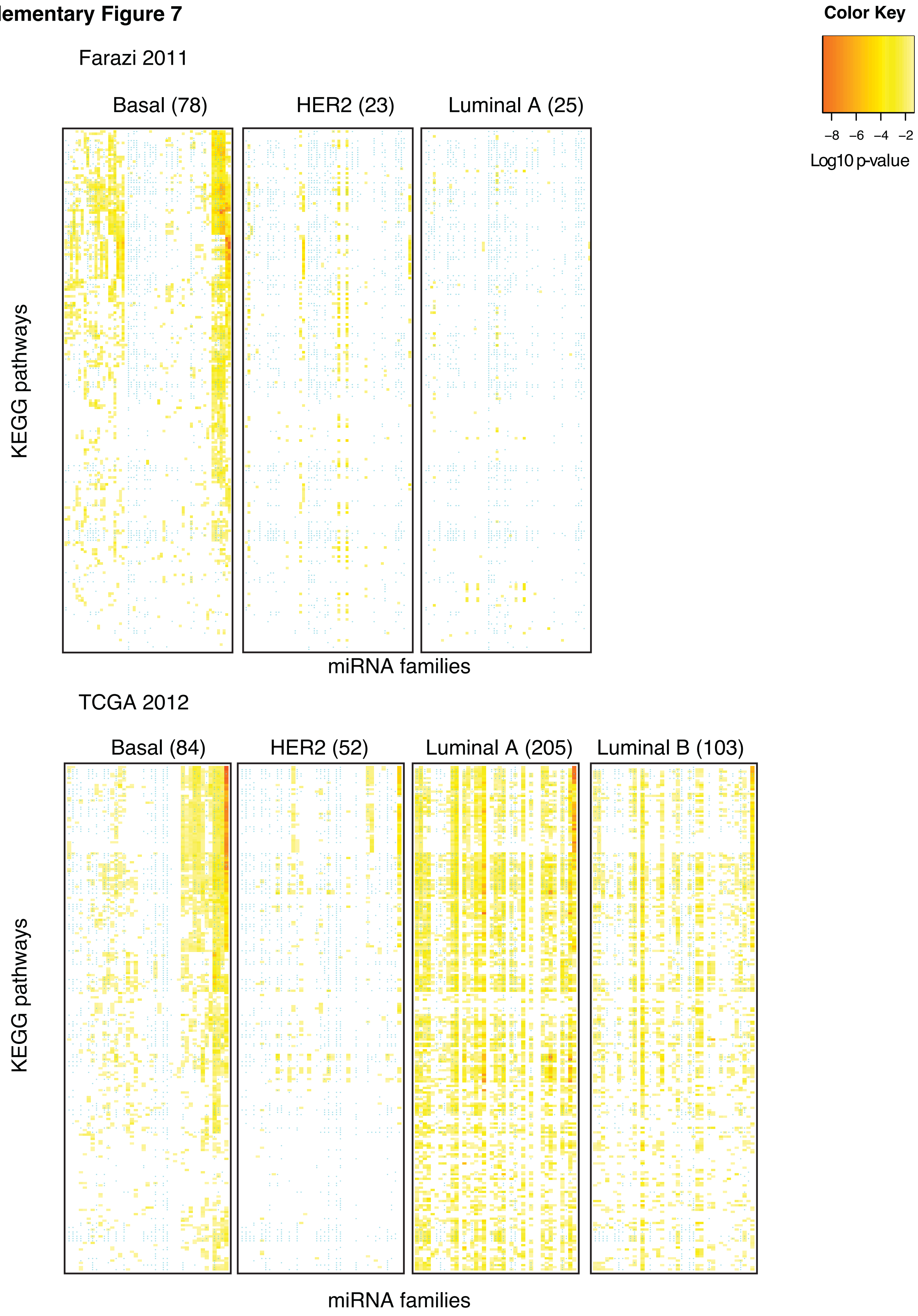

Supplementary Figure 8A **Leukocyte transendothelial migration pathway** **Farazi 2011**

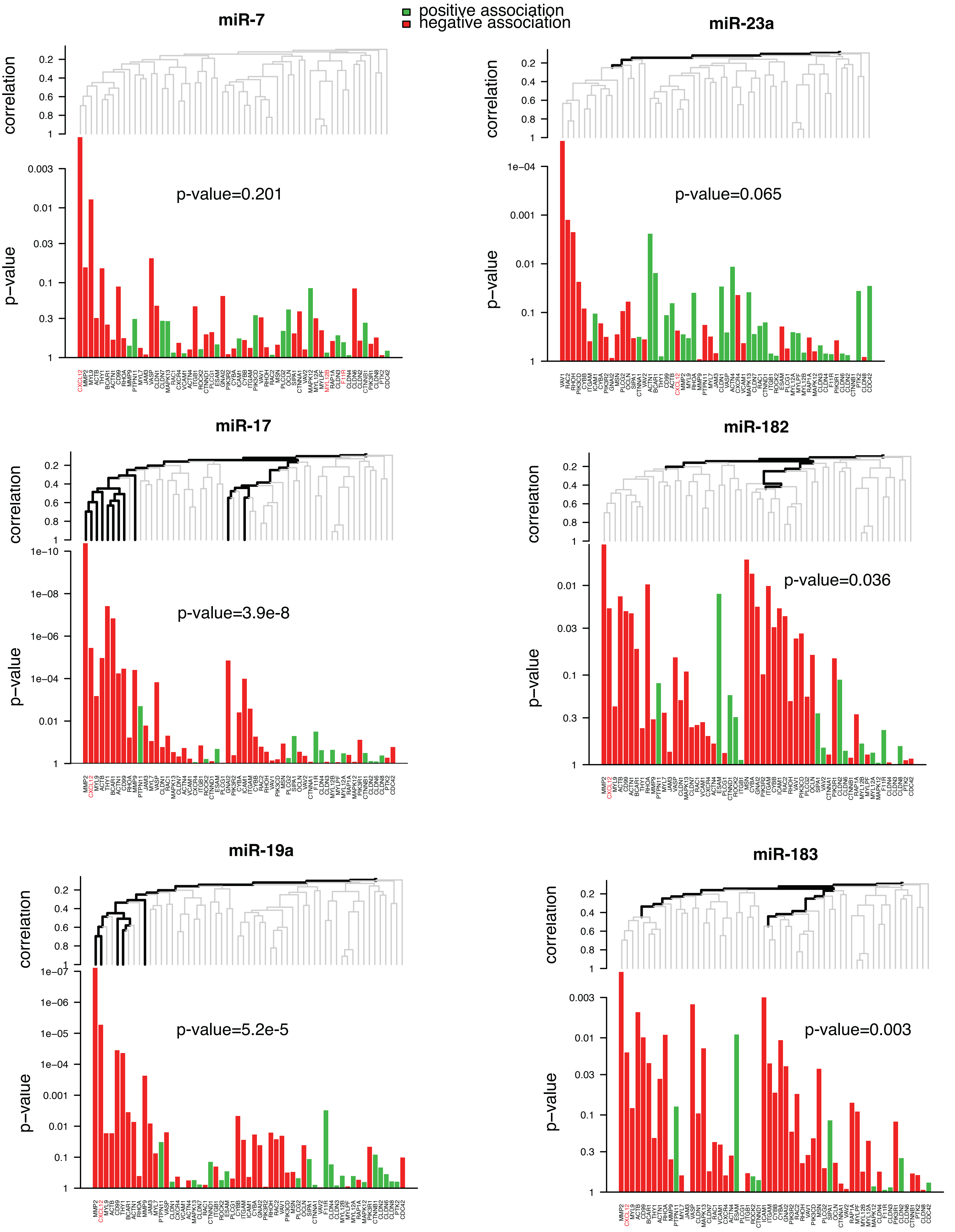

Supplementary Figure 8B

CXCL12 PAR-CLIP clusters

slc2890_chr10 — miR-7

```
AACAATCAGCTCCTTCCTGGAGACTGCCCAGCTAAAGCAATATGCATTTAAATACAGTCTTCCATTTGCAAGGGAAAAGTCTCTTGTAATCCGAATCTCT  SEQUENCE ID  #Copies
---------------------------------------------CATTCAAATACAGTCTTCCATTTG-------------------------------    84456548     3
---------------------------------------------CATTTAAATACAGTCTTCCATCTG-------------------------------    84438192     2
---------------------------------------------CATCTAAATACAGTCTTCCATTT--------------------------------    84450990     1
--------------------------------TAAAGCAATATGCACTTAAATACAG-------------------------------------------    83920003     1
---------------------------------------------CATTTAAATACAGTCTTCCACTTG-------------------------------    84438191     1
----------------------------------------------ATTTAAATACAGTCTTCCATCTG-------------------------------    84386723     1
```

slc2888_chr10 — miR-23a

```
GATGAATACTGTATTTTGTATTGTTTCAATTGCATCTCCCAGATAATGTGAAAATGGTCCAGGAGAAGGCCAATTCCTATACGCAGCGTGCTTTAA      SEQUENCE ID  #Copies
--------------------------------CATCTCCCAGACAATGTGAAAATG----------------------------------------        84451532     28
--------------------------------CATCTCCCAGACAATGTGAAAAT-----------------------------------------        84451531     18
--------------------------------CATCTCCCAGATAATGTGAAAATG----------------------------------------        84451539     3
--------------------------------CATCTCCCAGACAATGTGAAAA------------------------------------------        84451529     2
--------------------------------CATCTCCCAGACAATGTGAAAATGGTCCAGG---------------------------------        84451533     2
--------------------------------CATCTCCCAGATAATGTGAAAAT-----------------------------------------        84451538     1
--------------------------------CATCTCCCACATAATGTGAAAATG----------------------------------------        84451525     1
-----------------------------------CTCCCAGACAATGTGAAAAT-----------------------------------------        84268660     1
----------------------------------TCTCCCAGACAATGTGAAAATG----------------------------------------        83693094     1
--------------------------------CATCTCCCAGACAATGTGAAAATGGTCCAGGA--------------------------------        84451534     1
```

slc2898_chr10 — miR-182 — miR-19a, miR-17, miR-183

```
CACAGCCTCCCCTGCCAGGGCAGGGCCCCAGGCATTGCCAAGGGCTTTGTTTTGCACACTTTGCCATATTTTCACCATTTGATTATGTAGCAAAATACATGACATTTATTTTT    SEQUENCE ID  #Copies
--------------------------------CATTGCCAAGGGCTTTGCTTTGCACACTTTG--------------------------------------------------      84437773     2
------------------------------------------------------CACACTCTGCCATATTTTCACCATTTG--------------------------------      84406463     1
------------------------------------------------------CACACTTTGCCATATTCTCACCATTTG--------------------------------      84406664     1
--------------------------------CATTGCCAAGGGCTTTGTTTTGCACACTTTG--------------------------------------------------      84437774     1
```

PLCG1 PAR-CLIP clusters

slc789_chr20 — miR-200b

```
ATGACCCAGCCTGAAAGATACAGGGGATCATGTTAAAAATAGCAGTATTATTTTTCGTCTCAATGGTATTGTAACTAAGTTATTTACTCCTCCTGCT    SEQUENCE ID  #Copies
--------------------------------TTAAAAACAGCAGTATTATTTTTCG----------------------------------------      84021361     10
--------------------------------TTAAAAATAGCAGTATTA-TTTTCG----------------------------------------      57016491     4
--------------------------------TTAAAAATAGCAGTATTATTTCTCG----------------------------------------      84028104     3
--------------------------------TTAAAAATAGCAGTATTACTTTTCG----------------------------------------      84028042     3
--------------------------------TTAAAAATAGCAGTATTATTTTTCG----------------------------------------      60382777     2
--------------------------------TTAAAAATAGCAGTATTATCTTTCG----------------------------------------      84028070     2
--------------------------------TTAAAAATAGCAGTATTATTCTTCG----------------------------------------      57016483     2
--------------------------------TTAGAAATAGCAGTATTATTTTTC-----------------------------------------      84024100     1
--------------------------------TTAAAAATAGCAGTATTATTTCTC-----------------------------------------      84028093     1
--------------------------------TTAAAAATAGCAGTATTATTTTCCGTCTCAATG--------------------------------      84028126     1
--------------------------------TTAAAAATAGCAGTATTATTTTTC-----------------------------------------      84028153     1
--------------------------------TTAAAAATAGCAGTATTATTTT-------------------------------------------      84028115     1
--------------------------------TTAAAAATAGCAGTATTATTTTT------------------------------------------      84028143     1
--------------------------------TTAAAAATAGCAGTATT-TTTTTC-----------------------------------------      84028171     1
```

Supplementary Figure 8C **Farazi 2011**

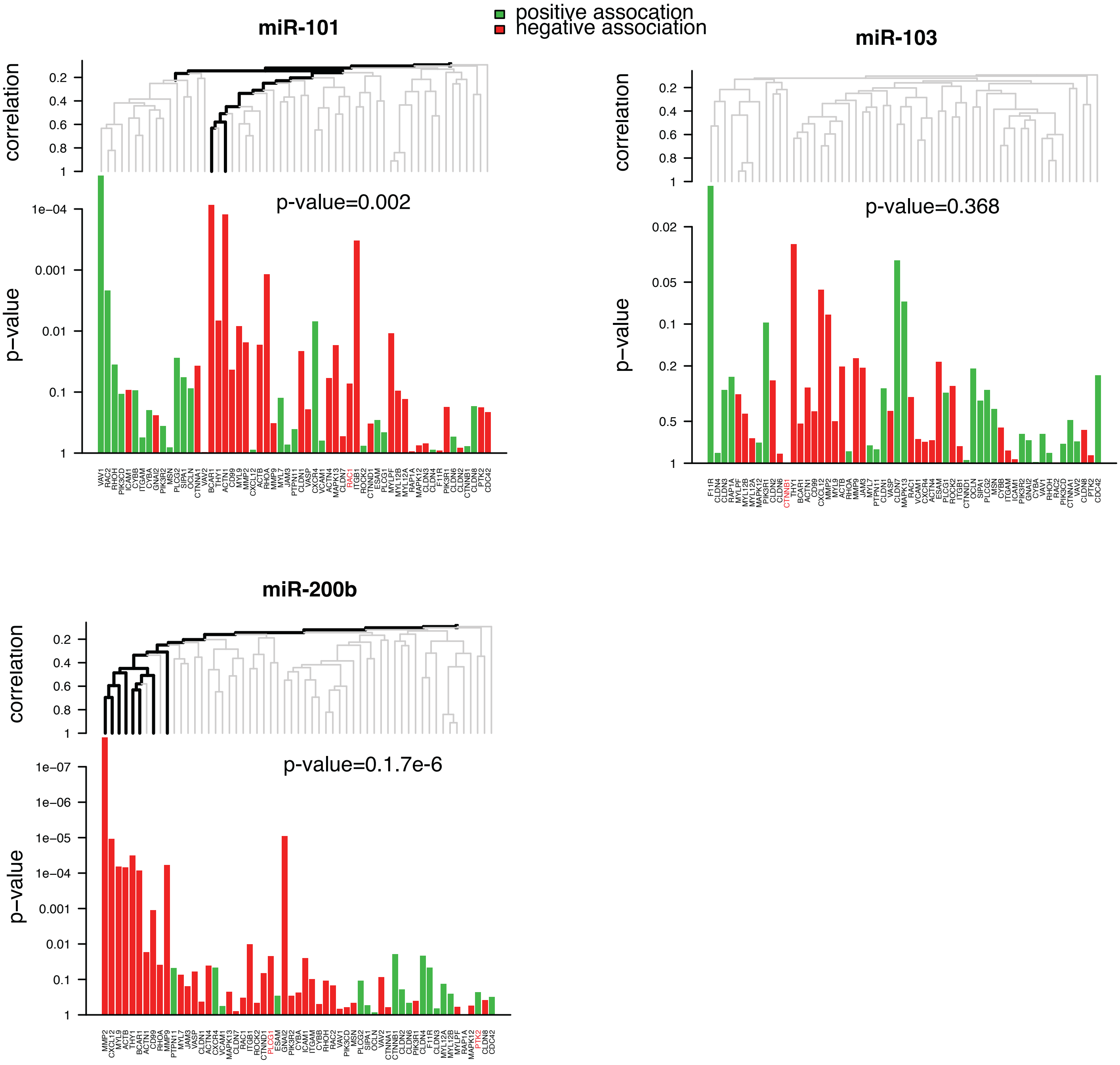

Leukocyte transendothelial migration pathway

Supplementary Figure 8D **TCGA 2012**

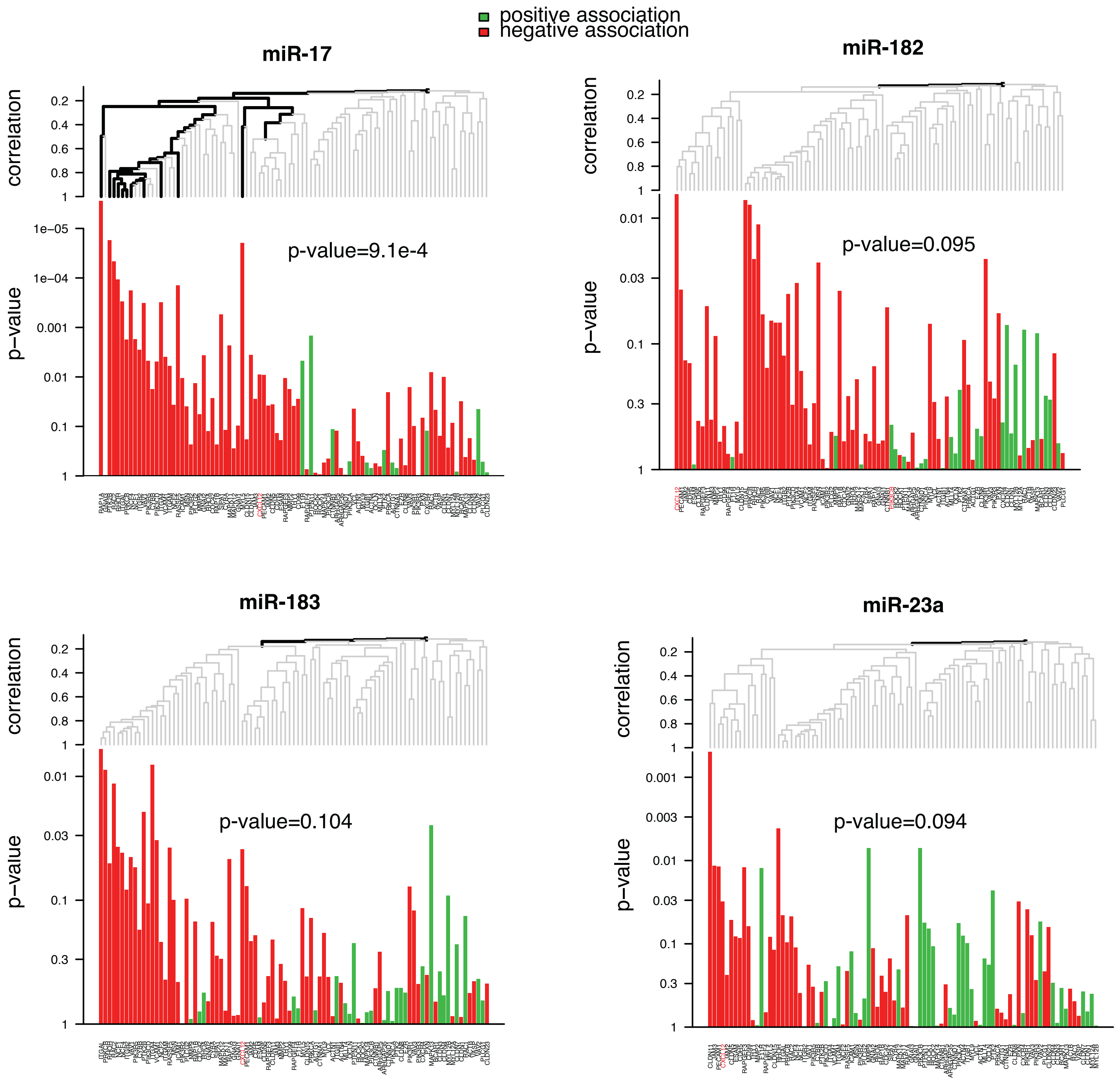

Leukocyte transendothelial migration pathway

Supplementary Figure 9

DATA used for miRNA-target identification

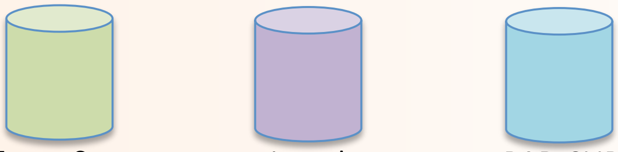

TargetScan   Patient data   PAR-CLIP

1.

REGRESSION MODEL to rank additional miRNA targets

2. →

miRNA	Gene	# conserved sites	...	Total context score	Aggregate PCT	miRNA expression	miRNA variance	mRNA expression	mRNA variance	Interaction term 1	Interaction term 2	PAR-CLIP target		Prediction
miR-30abcdef...	CELSR3	5	...	-0.551	1.000	0.031	2.70E-04	-0.078	0.043	-0.002	0.006	1		0.75
miR-23abc/23...	KIAA1467	3	...	-0.764	0.927	0.022	1.33E-04	-0.358	0.069	-0.008	0.009	1		0.74
miR-29abcd	TET2	4	...	-0.735	0.999	0.023	1.09E-04	0.354	0.410	0.007	0.014	0		0.74
let-7/98/4458...	SERF2	1	...	-0.545	0.854	0.109	0.002	-0.153	0.095	-0.015	0.031	0	Training set	0.72
miR-125a-5p/...	STARD13	2	...	-0.959	0.982	0.020	2.69E-04	-0.020	0.077	2.68E-04	0.004	0		0.68
...	...	...	...	...	...	...	...	...	...	...	...	...		...
miR-34ac/34bc...	HCN3	4	...	-1.667	0.998	0.001	1.12E-06	0.025	0.052	5.59E-05	2.33E-04	0		0.96
miR-21/590-5p	WWP1	1	...	-0.209	0.299	0.312	0.014	-1.381	0.406	-0.418	0.431	0		0.88
let-7/98/4458/...	COL1A2	1	...	-0.411	0.954	0.109	0.002	-1.243	1.150	-0.148	0.160	0		0.88
miR-199ab-5p	C11orf9	3	...	-0.991	0.998	0.008	8.04E-05	-0.136	0.084	-0.001	0.002	0		0.80
let-7/98/4458...	PRTG	3	...	-0.557	0.999	0.109	0.002	-0.189	0.055	-0.022	0.027	0		0.78
miR-29abcd	COL3A1	2	...	-0.929	0.979	0.023	1.09E-04	-0.948	1.124	-0.025	0.028	0		0.78
miR-145	TPM3	2	...	-1.063	0.855	0.009	7.21E-05	-0.169	0.154	-0.002	0.003	0		0.75
miR-148ab-3p...	OSBPL11	2	...	-0.888	0.960	0.027	1.84E-04	0.261	0.150	0.007	0.010	0	Discovery set	0.75
miR-145	FSCN1	4	...	-0.973	0.997	0.009	7.21E-05	0.028	0.471	-0.001	0.004	0		0.74
miR-181abcd/...	PRTG	5	...	-0.661	0.991	0.005	4.56E-05	-0.189	0.055	-0.001	0.001	0		0.70
miR-103a/107...	NF1	2	...	-0.849	0.873	0.016	3.95E-05	-0.627	0.313	-0.010	0.011	0		0.69
miR-27abc/27...	PLK2	3	...	-0.748	0.970	0.015	6.61E-05	-0.166	0.540	-0.002	0.008	0		0.67
let-7/98/4458...	HAND1	1	...	-0.437	0.985	0.109	0.002	0.120	0.039	0.013	0.020	0		0.67
...	...	...	...	...	...	...	...	...	...	...	...	...		...

3.

PRIORITIZATION of miRNA regulatory activity and phenotype association

miRNA	Target effect	Pathway activity	Cancer association	RANK 1	Metastasis	Survival	Histological grade	Lymphangio invasion	Positive lymph nodes	Tumor size	RANK 2
miR-17/17-5p/20ab/...	4.54E-08	2.29E-11	8.88E-07	1	0.580	0.687	0.116	0.007	0.286	0.476	**2**
miR-200bc/429/548a	4.42E-05	3.97E-09	2.95E-06	2	0.578	0.794	0.252	0.155	0.429	0.536	**5**
miR-25/32/92abc/363...	0.001	2.77E-09	2.99E-07	3	0.298	0.257	0.254	0.019	0.550	0.750	**11**
miR-19ab	2.80E-04	2.17E-07	2.43E-05	4	0.281	0.613	0.094	0.065	0.363	0.032	**1**
...	...	...	...	**...**	...	...	...	...	...	...	**...**
miR-145	0.065	2.11E-06	0.009	**45**	0.348	0.842	0.514	0.063	0.401	0.782	**6**
miR-29abcd	0.016	2.73E-04	0.007	**46**	0.098	0.601	0.113	0.055	0.124	0.966	**7**
let-7/98/4458/4500	0.155	0.011	0.358	**50**	0.337	0.837	0.256	0.005	0.265	0.276	**52**
miR-7/7ab	0.001	0.001	0.045	**51**	0.139	0.478	0.426	0.038	0.337	0.915	**40**
miR-22/22-3p	0.170	0.001	0.004	**52**	0.549	0.711	0.077	0.116	0.363	0.613	**22**

4.

VALIDATION in independent patient datasets

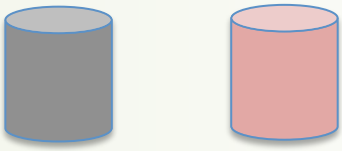

TCGA   NKI295

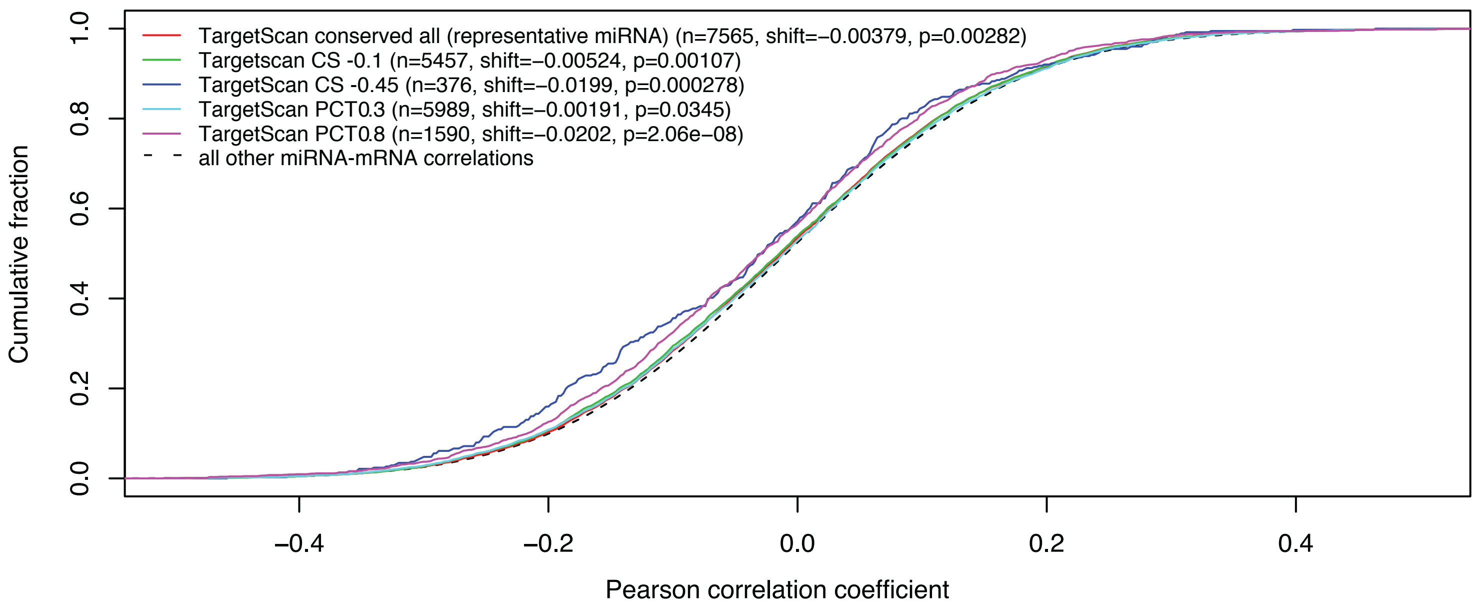
TargetScan conserved all (representative miRNA) (n=7565, shift=−0.00379, p=0.00282)
Targetscan CS -0.1 (n=5457, shift=−0.00524, p=0.00107)
TargetScan CS -0.45 (n=376, shift=−0.0199, p=0.000278)
TargetScan PCT0.3 (n=5989, shift=−0.00191, p=0.0345)
TargetScan PCT0.8 (n=1590, shift=−0.0202, p=2.06e−08)
all other miRNA-mRNA correlations
Cumulative fraction
0.0
0.2
0.4
0.6
0.8
1.0
−0.4
−0.2
0.0
0.2
0.4
Pearson correlation coefficient

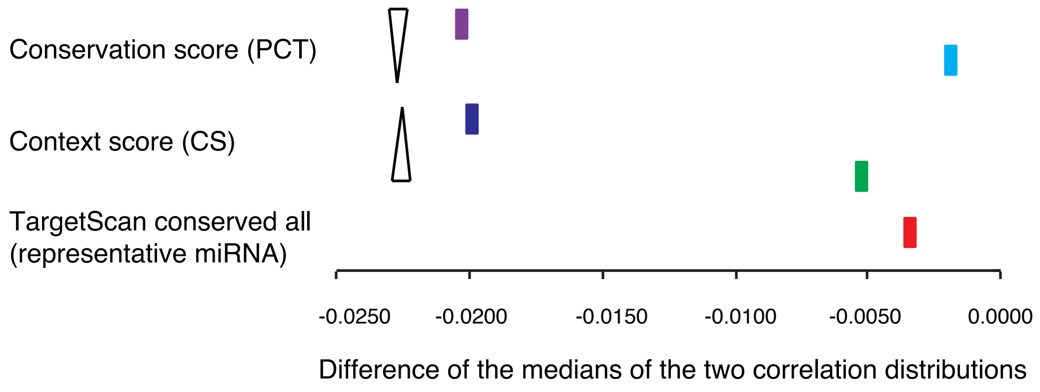
Conservation score (PCT)
Context score (CS)
TargetScan conserved all
(representative miRNA)
-0.0250
-0.0200
-0.0150
-0.0100
-0.0050
0.0000
Difference of the medians of the two correlation distributions

Supplementary Figure 11 **Farazi 2011**

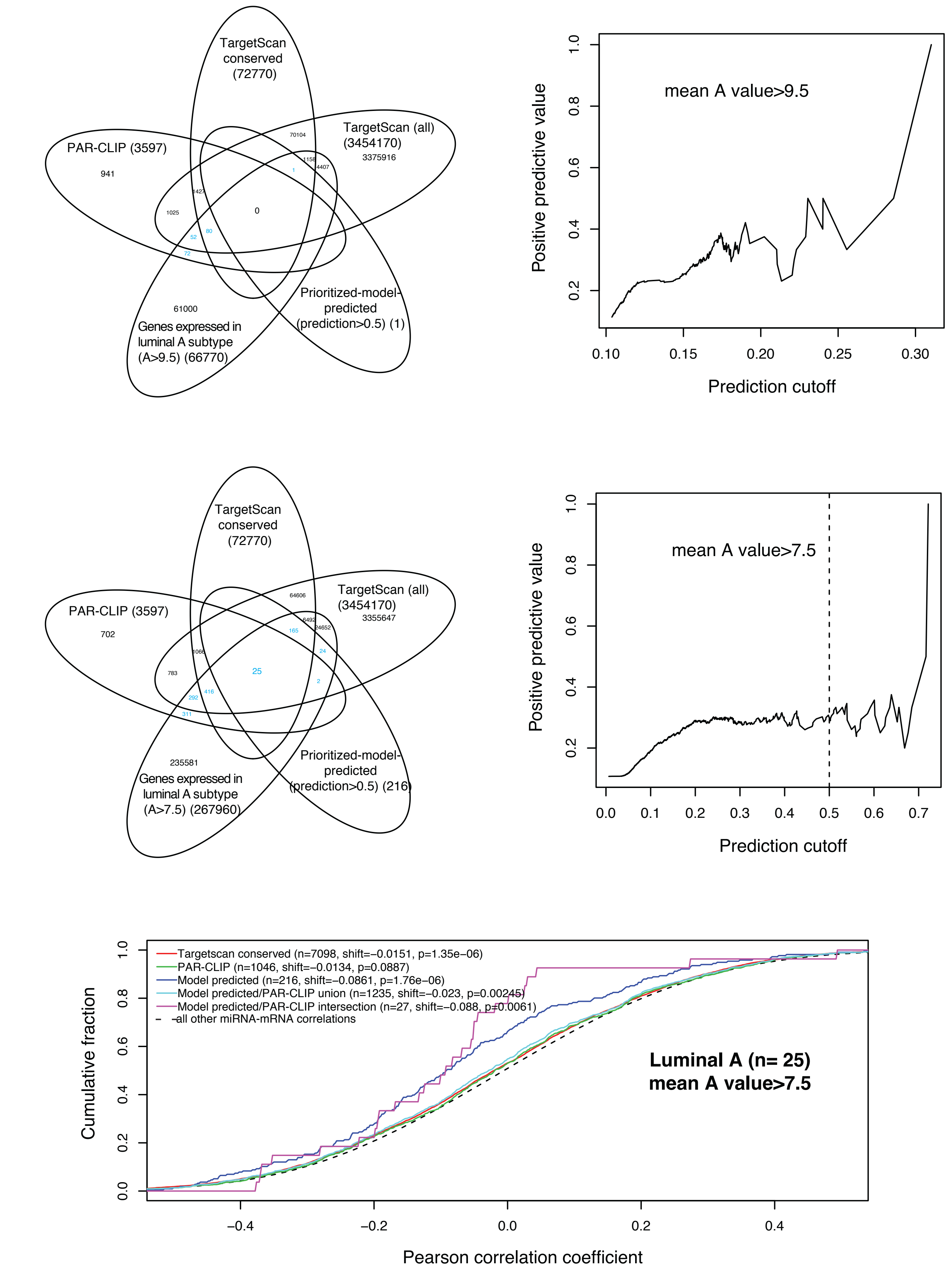

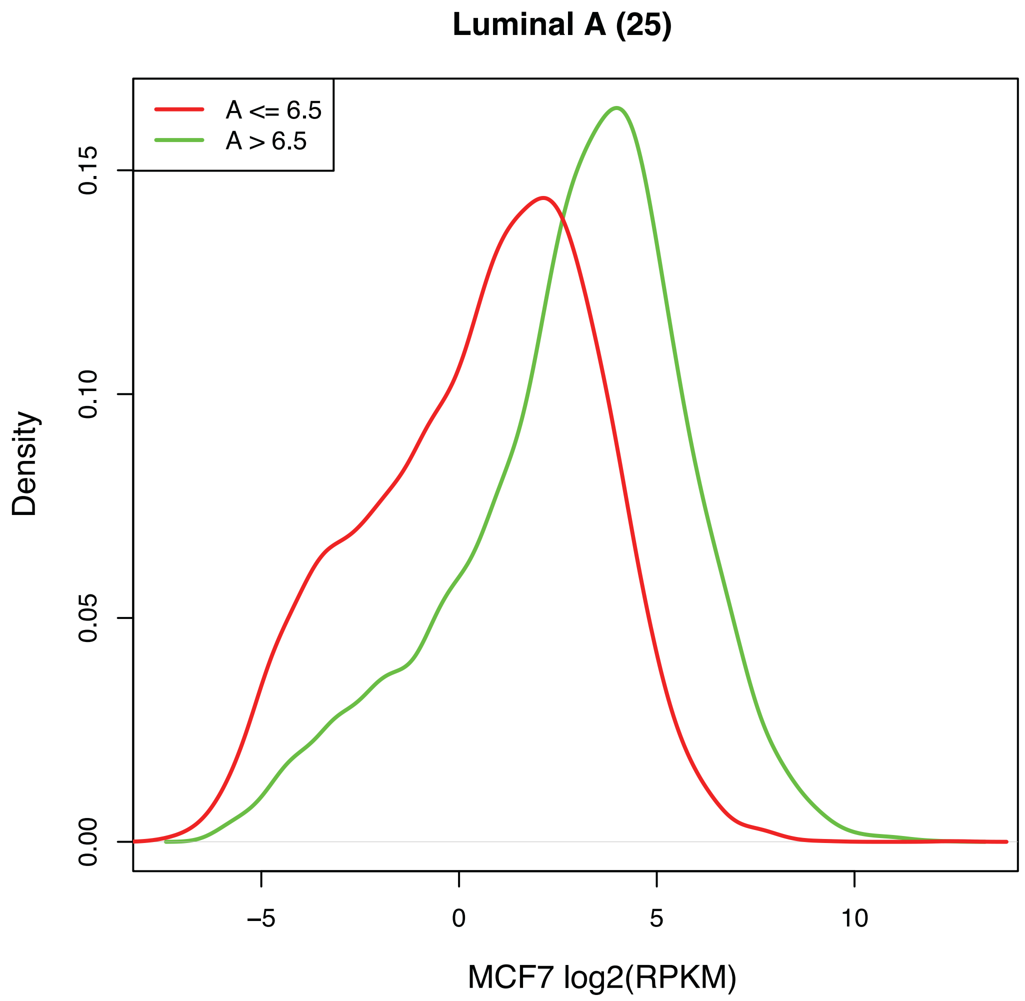
Luminal A (25)
A <= 6.5
A > 6.5
Density
0.00
0.05
0.10
0.15
−5
0
5
10
MCF7 log2(RPKM)

Supplementary Figure Legends

Supplementary Figure 1: MiRNA and mRNA abundance thresholds in patient datasets. A and B. Pearson correlation between expression of intronic miRNAs and host genes. Cumulative fraction of Pearson correlation between expression of intronic miRNAs (RRF) and their host genes (log_2(fold-change)); normalized to a pool of 100 tumors) (solid line) compared to the correlation between the remainder miRNA-mRNA pairs (dashed line). Analysis conducted using 161 samples from ([15]) (A) and 444 samples from [13] (B). **C and D. Dependence of Pearson correlation between expression of intronic miRNAs and host genes on miRNA and mRNA abundance in 161 samples [15].** Black dots represent correlations between expression of intronic miRNAs and their host genes; grey dots represent correlations between the remainder miRNA-mRNA pairs. Black and grey lines depict the regression coefficients (associated p-values shown below). **E-H. Dependence of Pearson correlation between expression of miRNA families and their conserved TargetScan-targets on selected threshold for mRNA and miRNA abundance in 161 samples [15].** Dependence of difference (and Wilcoxon-rank-sum-test p-value) of the medians of the distribution of miRNA-TargetScan-target correlations compared to the distribution of the remainder miRNA-mRNA correlations on selected threshold for mRNA (E-F) or miRNA abundance (G-H). **I. The difference of the medians of the distribution of miRNA-target correlations compared to the distribution of the remainder miRNA-mRNA correlations for each molecular subtype for [15].** Results shown for two thresholds of mRNA abundance.

Supplementary Figure 2: MiRNA and mRNA abundance thresholds in patient datasets. A and B. Dependence of Pearson correlation between expression of intronic miRNAs and host genes on miRNA and mRNA abundance in 444 samples [13]. Black dots represent correlations between expression of intronic miRNAs and their host genes; grey dots represent correlations between expression of the remainder miRNA-mRNA pairs. Black and grey lines depict the regression coefficients, with associated p-values shown below. **C-F. Dependence of Pearson correlations between expression of miRNA families and their conserved TargetScan-targets on selected threshold for mRNA and miRNA abundance.** Dependence of difference of the medians of the distribution of miRNA-TargetScan-target correlations compared to the distribution of the remainder miRNA-mRNA correlations, and its associated Wilcoxon-rank-sum-test p-value, on selected threshold for mRNA (C-D) or miRNA abundance (E-F).

Supplementary Figure 3: miRNA-target correlation analysis using MiRanda target predictions. A. Dependence of the difference of the medians of the distribution of miRNA-MiRanda-target correlations compared to the distribution of the remainder miRNA-mRNA correlations on miRSVR score, and comparison with conserved miRNA-TargetScan-target correlations used for analysis in this manuscript. **B-C.** Dependence of Pearson correlations between expression of miRNAs and their MiRanda-targets (miRSVR cutoff<-1.2) on selected threshold for mRNA abundance. Dependence of difference of the medians of the distribution of miRNA-MiRanda-target correlations

compared to the distribution of the remainder miRNA-mRNA correlations, and its associated Wilcoxon-rank-sum-test p-value, on selected threshold for mRNA abundance.

Supplementary Figure 4: Correlation analysis by molecular subtypes. Correlation between expression of miR-17 family and expression of its respective top ten anti-correlated conserved TargetScan-targets (among all samples) across molecular subtypes in [15] and [13]. Anti-correlated genes selected from [15] (A) or [13] (B).

Supplementary Figure 5: miRNA target model prediction in the luminal A subtype in our dataset [15]. A and B. Venn diagrams depicting overlap of associations between miRNA family and TargetScan conserved/non-conserved/PAR-CLIP identified/model-predicted targets with miRNA and mRNA expression in patient samples (based on our miRNA and mRNA abundance thresholds). Training set associations in red represent false interactions (between combinations of non-site-seed-matched miRNA-mRNA pairs identified in PAR-CLIP) and associations in green represent true interactions (between site-seed-matched PAR-CLIP miRNA-mRNA pairs). MP-PCLIP targets selected for downstream analysis are shown in blue. **C.** Regression model positive predictive value as a function of selected posterior probability score threshold on the left; AUC plot on the right. **D.** Correlation distribution of expression of miRNA families and their conserved TargetScan, PAR-CLIP identified and model-predicted targets compared to the correlation distribution of expression of all other miRNAs and mRNAs. **E-G.** Correlation distribution of expression of miRNA families and their conserved TargetScan, PAR-CLIP identified and model-predicted targets compared to the correlation distribution of

expression of all other miRNAs and mRNAs in the basal-like, HER2 and luminal B subtypes.

Supplementary Figure 6: miRNA target model prediction in the luminal A subtype in the TCGA dataset [13]. Same analysis as in Supplementary Fig. S5.

Supplementary Figure 7: miRNA KEGG pathway associations. Heatmaps depicting significant p-values from global tests correlating expression of miRNA families to genes belonging to KEGG pathways for different subtypes in [15] and [13]. Heatmaps for HER2, luminal A and luminal B subtype are ordered according to the clustering of the basal-like subtype for each dataset. Heatmap for luminal A subtype in [15] did not include any significant p-values.

Supplementary Figure 8: Association of miRNA family expression with the leukocyte transendothelial migration pathway. A. Global test associations between expression of genes belonging to the leukocyte transendothelial migration pathway and expression of the depicted miRNA families targeting CXCL12 in our dataset [15]. The height of each bar represents the p-value for the association of each gene and miRNA family, and the color represents the type of association (red, negative; green, positive). Unsupervised hierarchical clustering of genes is based on their correlation values with miRNA expression. All 6 miRNAs target CXCL12, as evidenced by PAR-CLIP. CXCL12 and additional miRNA targets are depicted in red. **B.** PAR-CLIP clusters corresponding to miRNA binding sites on CXCL12. miRNA seed sequence is depicted in

red, T to C conversions are depicted in green. **C.** Global test associations between expression of genes belonging to the leukocyte transendothelial migration pathway and the expression of the depicted miRNA families, highlighting miRNAs with targets other than CXCL12. MiRNA targets are depicted in red. **D.** Same as panel A for the TCGA dataset.

Supplementary Figure 9: Overview of analysis and tables generated. 1. Types of data used for miRNA-target identification in patient samples. The data was filtered based on miRNA/mRNA abundance in patients within each molecular subtype. 2. Regression model to rank additional subtype-specific miRNA targets based on TargetScan prediction, PAR-CLIP and patient data characteristics. 3. Correlation and GT analysis to prioritize miRNA regulatory activity and associated tumor phenotype. 4. Validation of top candidates in independent datasets.

Supplementary Figure 10: miRNA-target correlation analysis using thresholds for TargetScan context and conservation score in our dataset. Comparison of correlation distribution of expression of representative miRNAs from each TargetScan family and their conserved targets to the correlation distribution of expression of all other miRNAs and mRNAs. The figure illustrates the effect of different context score and conservation score thresholds on the magnitude of the shift, comparing each correlation distribution to the correlation distribution of all other miRNAs and mRNAs.

Supplementary Figure 11: miRNA target model prediction in the luminal A subtype in our dataset [15] using more stringent mRNA abundance thresholds. Same analysis as in Supplementary Fig. S5.

Supplementary Figure 12: The distribution of low ($A<6.5$) and high ($A>6.5$) expressed genes in the patient luminal A samples [15] as a function of the MCF7 cell RPKM expression levels.

Supplementary Table Legends

Supplementary Table 1: Summary of published studies.

Supplementary Table 2: Correlation of ranking of miRNA targets between the Farazi 2011 and TCGA 2012 datasets for each individual miRNA within distinct molecular subtypes.

Supplementary Table 3: AGO2-PAR-CLIP supplementary data. A. AGO2-PAR-CLIP sequence mapping statistics. **B.** MCF7 miRNA expression profile for mature miRNAs on the left. AGO2-PAR-CLIP associated miRNAs and their T-to-C conversion frequency on the right. **C.** AGO2-PAR-CLIP enrichment table. Mir: name of mature miRNA; miRNA seed (1-6, 1-7, 1-8, 1-9, 1-10, 2-7, 2-8, 2-9, 2-10, 3-8, 3-9, 3-10, 4-9, 4-10); cluster count: number of clusters containing specified seed; background mean and Std Dev: expected background frequency and standard deviation of k-mer of same length as miRNA seed; p-value: describes likelihood of seed enrichment of top miRNA k–mer seed sequence to its expected background frequency. **D.** AGO2-PAR-CLIP miRNA-mRNA targets identified based on seed sequence complementarity for 7-mer 2-8. CCR: Name of CCR based on its location in the genome; sequence: 41 nucleotide CCR sequence; seed hit: 7-mer seed sequence for which search was conducted; position found: position in CCR; miRNA: name of mature miRNA; cluster count: number of clusters identified containing seed sequence; Gene ID: gene symbol; gene description; Type of seed: 1-7 (allowing A mismatch at position 1) or 2-8.

Supplementary Table 4: Regression model to predict miRNA 'PAR-CLIP-like' targets in patient datasets. Univariate correlation analysis using a combination of LASSO and ridge regression to define features of AGO2-PAR-CLIP targets. Rows list each individual TargetScan and patient miRNA-mRNA expression profile characteristic. Columns list: univariate correlation; univariate correlation p-value; univariate AUC; selection by LASSO regression model (true or false).

Supplementary Table 5: T-test comparing p-values of miRNA-pathway associations that include at least one MP-PCLIP miRNA target compared to p-values of all other miRNA-pathway associations for the basal-like subtype in our dataset.

Supplementary Table 6: Ranking lists for miRNA phenotype association and target prognostic signatures. A and B. Ranking miRNA phenotype for the Farazi and NKI295 datasets. See Results and Materials and Methods for details. **C through E.** Benjamini-Hochberg corrected p-values for global test Cox-regression for metastasis and overall survival of validated miRNA targets for the Farazi, NKI295 and combined breast cancer datasets. Also listed is the number of genes in each prognostic gene set.

Supplementary Table 7: Intronic miRNAs and their host genes whose expression shows positive correlation. The table lists the Pearson correlation, miRNA (RRF) and mRNA (A value or log_2RPKM) abundance for our dataset and the TCGA dataset, respectively.
